# Direct laser writing of electronically conductive microstructures within soft hydrogel substrates

**DOI:** 10.1016/j.mtbio.2025.102140

**Published:** 2025-07-30

**Authors:** Lorenzo Lucherini, Veronica Navello, Outman Akouissi, Stéphanie P. Lacour, Esther Amstad

**Affiliations:** a Soft Materials Laboratory, Institute of Materials, École Polytechnique Fédérale de Lausanne, Lausanne, Switzerland; b Laboratory for Soft Bioelectronic Interfaces, Neuro X Institute, École Polytechnique Fédérale de Lausanne (EPFL), Geneva, Switzerland; c Bertarelli Foundation Chair in Translational Neuroengineering, Neuro X Institute, École Polytechnique Fédérale de Lausanne (EPFL), Geneva, Switzerland

## Abstract

Hydrogels have emerged as promising materials for bioelectronic interfaces due to their tissue-like properties and high-water content. However, conventional hydrogels often suffer from poor electrical conductivity and mechanical stability, limiting their performance in long-term bioelectronic applications. Electronic conductivity can be imparted to hydrogels by functionalizing them with conductive particles. However, patterning of electronically conductive features within hydrogels remains challenging. Electronically conductive μm-sized patterns embedded in soft hydrogels would open up new possibilities to integrate hydrogel bioelectronics with electronic devices. Here, we introduce covalently crosslinked hydrogels with Young's moduli below 30 kPa that can be functionalized with metallic electronically conductive paths reaching an electronic conductivity up to (1505 ± 518) S cm^−1^. By tailoring the hydrogel substrate composition, we achieve writing fidelity up to ±5 %, with feature width as narrow as 5 μm. Using two-photon direct laser writing, we demonstrate the ability to pattern encapsulated conductive structures at the surface or within the bulk of the hydrogels. These patterned hydrogels offer new opportunities for creating soft, miniaturized bioelectronic interfaces, with potential applications in cellular and tissue electrophysiology.

## Graphical abstract

**Figure:**
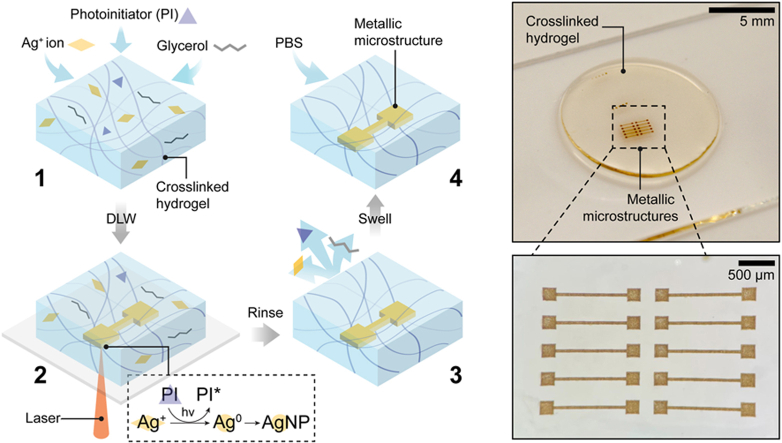

## 1 Introduction

Rapid advancements in soft bioelectronics call for engineered materials that integrate low elastic moduli and compliance with electrical conductivity, thereby creating a seamless interface between biological tissues and artificial devices [1,2]. Hydrogels are hydrophilic polymeric networks with high water content, low Young's moduli (<100 kPa), and ionic conductivity and hence, they mimic many aspects of natural soft tissues. Indeed, biocompatible hydrogels are often used in implantable soft bioelectronics [[3], [4], [5], [6]], wearable electronics [[7], [8], [9], [10]], and tissue engineering [[11], [12], [13], [14]]. These applications demand for conductivity within the hydrogel for the recording or stimulation of electrophysiological signals. The high water content of hydrogels enables incorporation of high concentrations of ions that confer ionic conductivity [1,15]. Yet, to record electrophysiologic signals and stimulate tissues, ion concentrations in the molar range are needed [1,16]. These ion concentrations are significantly higher than physiological levels, causing ions to diffuse out of the hydrogel upon exposure to physiological environment. This ion out diffusion alters the hydrogel conductivity over time, ultimately compromising the stability and reliability of measurements [16]. To overcome this limitation, hydrogels can also be rendered electronically conductive through their functionalization with conductive additives including metallic [7,[17], [18], [19]] and carbon fillers [3,11,12] that form a percolation network or intrinsically conductive polymers such as poly(3,4-ethylenedioxythiophene) (PEDOT) [6,[20], [21], [22], [23], [24]]. If electronically conductive hydrogels also contain free ions, electronic and ionic conductivity is combined, resulting in hydrogels with mixed ionic-electronic conductivity, which can be characterized by means of electrochemical impedance spectroscopy [20,[25], [26], [27]]. Electronically conductive additives are typically homogeneously distributed within the hydrogels, forming bulk materials. Spatially-resolved stimulation and recording of electrophysiological signals from soft tissues would require conductive additives to be localized within pre-defined regions [3,6,28]. Localized electronically conductive paths can be introduced into soft substrates such as elastomers through patterning technologies including photolithography, metal evaporation and sputtering [4,[28], [29], [30]]. Unfortunately, these processes cannot be readily used to pattern hydrogels because photoresist processing entails exposures to high temperatures and metal deposition is performed at low pressures [31,32]. To circumvent these limitations, hydrogels have been functionalized with electronically conductive paths through extrusion-based three-dimensional (3D) printing [6,10,20,33], embedded 3D printing [17,34], and mold-casting [3,7,24,35]. The writing resolution of these techniques is limited to 100 μm if the rheological properties of the conductive ink are optimized. Yet, in most cases, the writing resolution is significantly higher. Moreover, the incorporation of conductive fillers such as silver microparticles or conductive polymers significantly influences the rheological properties of 3D printing ink and the mechanical properties of the resulting material. Consequently, materials that simultaneously exhibit high electrical conductivity, mechanical and rheological properties suited for soft bioelectronics remain to be shown [17,20,36]. Photopatterning can reach lower resolutions, yet, is limited to writing planar structures on the substrate surface [12,31,[37], [38], [39]].

Direct laser writing (DLW) based on two-photon absorption has emerged as a promising technique for creating 3D metallic structures reaching sub-micrometer resolution [[40], [41], [42], [43], [44], [45], [46], [47], [48], [49], [50], [51], [52], [53], [54]]. This approach relies on the localized photoreduction of metal ions, which leads to the nucleation and growth of metallic nanoparticles (NPs) that eventually form interconnected 3D structures. For example, 3D conductive metallic structures have been printed into gelatin that acted as sacrificial substrates [[40], [41], [42]]. However, the low gel-sol transition temperature of gelatin limits the laser power range used for DLW, as the laser-induced localized heating can partially trigger the gelatin gel-sol transition, potentially compromising the writing fidelity and resolution [40,55,56]. This limitation can be overcome with covalently crosslinked hydrogels. Covalently crosslinked hydrogels offer another benefit: their mechanical properties can be tuned to match those of biological tissues. Indeed, metallic NPs have been produced within covalently crosslinked hydrogels such as poly(hydroxyethyl methacrylate) (PHEMA) [43] and poly(ethylene glycol) diacrylate (PEGDA) [[44], [45], [46], [47], [48], [49]]. However, the concentration of nanoparticles was low such that they likely did not form a percolating structure required for achieving electronic conductivity [37,50]. Methods that enable the direct fabrication of μm-sized, electronically conductive 3D paths in covalently crosslinked hydrogels, which maintain their structural integrity after the DLW process has been completed, remain to be introduced. Such methods might open up new possibilities for designing fully hydrogel-based devices for soft bioelectronics and *in vitro* tissue engineering.

Here, we employ DLW to introduce electronically conductive μm-sized paths into a covalently crosslinked, antifouling hydrogel, poly-[2-(Methacryloyloxy)ethyl]dimethyl-(3-sulfopropyl)ammonium hydroxide (PDMAPS), whose stiffness can be tuned to be as low as (33 ± 4) kPa, similar to those of the spinal cord or the retina [5]. By optimizing the hydrogel composition and DLW process parameters, we introduce patterns into the covalently crosslinked hydrogels that reach a conductivity up to (1505 ± 518) S cm^−1^ in the wet state. Unlike methods that incorporate large amounts of pre-formed conductive fillers into the hydrogel that potentially alter its rheological properties, our approach synthesizes AgNPs *in situ* via a bottom-up process directly within the hydrogel, preserving the substrate rheological properties. We demonstrate the versatility of this method in patterning metallic structures with resolution down to 5 μm, both exposed on the hydrogel surface and embedded up to 50 μm within the hydrogel bulk. We envisage this technique to enable direct functionalization of a diverse set of hydrogels, including poly(acrylamide), widely adopted in biological applications. These hydrogels encompassing electronically conductive micropatterns could be used, for example, as bio-electrodes, and soft microelectrode arrays for stimulation or sensing of organoids and cell culture.

## 2 Results and discussion

### 2.1 Hydrogel substrate design for DLW

**Fig. 1:**
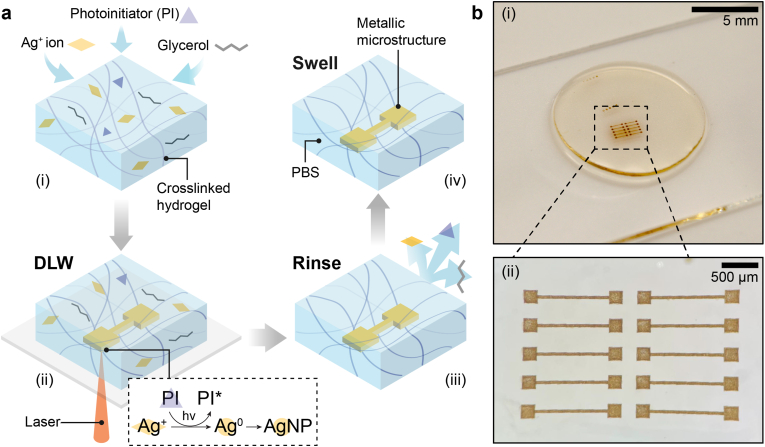
**Schematic illustration of the direct laser writing (DLW) of silver microstructures within a hydrogel substrate. a)** A crosslinked hydrogel is swollen in an aqueous solution containing silver ions (Ag^+^), glycerol, and a photoinitiator (PI) (i). Silver nanoparticles (AgNPs) are locally synthesized by illuminating well-defined volumes through a two-photon direct laser writing process to initiate the photoreduction of Ag^+^ (ii). Unreacted components are removed by rinsing with de-ionized water (iii). The silver microstructure-containing hydrogel is swollen in phosphate buffer saline (PBS) (iv). **b)** Photograph of the hydrogel substrate with embedded silver microstructures (i) and optical microscopy image showing a magnified view of the patterned microstructures (ii).

To enable direct laser writing (DLW) within hydrogels, they must be optically transparent and have a high swelling ratio such that they can be loaded with a high concentration of metal precursor ions. We use a covalently-crosslinked hydrogel substrate to ensure it maintains its integrity even if locally exposed to elevated temperatures caused by the photothermal effect of the two-photon DLW process. To avoid ion-induced substrate stiffening, we select a hydrogel that does not form strong complexes with metal ions. We employ a biocompatible zwitterionic hydrogel with anti-biofouling properties, commonly employed in soft bioelectronics, poly[2-(Methacryloyloxy)ethyl]dimethyl-(3-sulfopropyl)ammonium hydroxide (PDMAPS), as a model hydrogel [[57], [58], [59], [60]]. PDMAPS fulfills these requirements, as shown in Fig. S1a. We prepare bulk PDMAPS hydrogels from aqueous solutions containing DMAPS as monomer, N,N′-Methylenebisacrylamide (MBAA) as a crosslinker, and a photoinitiator. This solution is cast into a mold before it is polymerized through UV light exposure that initiates the free radical polymerization of DMAPS. To remove unreacted reagents, we wash the hydrogels in aqueous solutions for 48 h. The hydrogels are swollen in an aqueous solution containing glycerol that facilitates the DLW process, a photoinitiator (PI), and AgNO_3_, as shown in Fig. 1a(i). To pattern metallic microstructures within the substrate, we use two-photon DLW to trigger the PI-mediated photoreduction of Ag^+^ within the hydrogel, resulting in the localized nucleation and growth of silver nanoparticles (AgNPs) that agglomerate into micron-sized structures, as shown in Fig. 1a(ii), Fig. S2 and Movie M1. Unreacted moieties are removed by washing the hydrogel with deionized water, as schematically shown in Fig. 1a(iii) and supported with FTIR measurements shown in Fig. S3.

Supplementary data related to this article can be found online at https://doi.org/10.1016/j.mtbio.2025.102140

The following are the Supplementary data related to this article.

Video 2 Video 2

During the writing process of the metallic structure, the ion concentration within the hydrogel is reduced, potentially leading to a change in the degree of hydrogel swelling. The swelling-induced change in substrate dimensions risks damaging the written features. Because of the importance of the hydrogel swelling ratio, we quantify this parameter by measuring the swelling ratio in water with and without free ions. The volume of dried PDMAPS increases by 1000 % if immersed in an aqueous solution containing 1 M AgNO_3_. By contrast, dried PDMAPS swells by only 150 % if immersed in ion-free water, as shown in Fig. S1b. These results indicate that during the washing step, PDMAPS shrinks because the ion concentration is reduced. Yet, we cannot visually detect any shrinkage-induced damage on the written patterns, as shown in Fig. S4. We thus hypothesize that the shrinkage of PDMAPS could be beneficial to the formation of a percolative network of AgNPs, similarly to what has been reported for hydrogels that were rendered highly conductive by functionalizing them with silver microparticles upon partial dehydration [7,18]. To test our hypothesis, we perform the same experiments on a polyelectrolyte hydrogel composed of poly(2-Acrylamido-2-methylpropane sulfonic acid) (PAMPS), which swells upon reduction of the ion concentration: PAMPS swells to (431 ± 36) % of its dry diameter if immersed in water, whereas it only swells to (73 ± 11) % if immersed in an aqueous solution containing 1 M AgNO_3_, as shown in Fig. S5a and S5b**.** The washing-induced PAMPS swelling breaks the metallic features, as shown in Fig. S5c. These results suggest that washing-induced swelling is more damaging to the written features than washing-induced shrinkage. Because of the favorable ion-induced swelling of PDMAPS and its suitability for DLW, we use this material for the remainder of this study. The patterned substrate is soaked in phosphate buffer saline (PBS) to equilibrate it in an environment that mimics physiological conditions, as is typical for bioelectronic applications. The degree of swelling of PDMAPS increases from 175 % for hydrogels with the highest tested crosslinker concentration, 1.8 mol%, to 500 % for hydrogels with the lowest tested crosslinker concentration, 0.4 mol%, as shown in Fig. 1a(iv) and Fig. S6. Note that the swelling equilibrium is reached within 24 h for all tested samples, as shown in Fig. S6. The substrate remains optically transparent, such that the embedded metallic microstructures can be visualized with optical microscopy, as shown in Fig. 1b(i-ii).

An essential feature of hydrogel implementation in soft bioelectronics is achieving a good mechanical match between the gel and the tissues it is in contact with [1,16]. To reach stiffnesses in the range of 10 kPa, we employ a crosslinker concentration as low as 0.4 mol% to formulate hydrogels with a Young's modulus of (9.7 ± 2.6) kPa, as shown in Fig. S7. Moreover, PDMAPS hydrogels exhibit a viscoelastic behavior. The plateau storage modulus of these gels increases from 90 to ∼450 Pa if we increase the crosslinker concentration from 0.4 mol% to 1.8 mol%, as shown in Fig. S8. These values are similar to values reported for human brain [5].

**Fig. 2:**
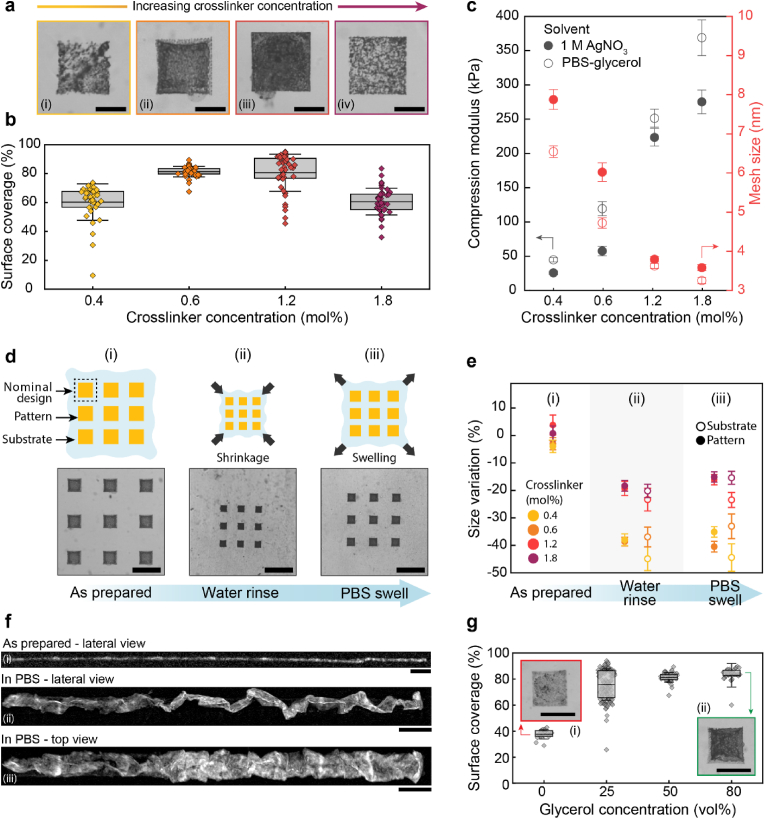
**Influence of hydrogel composition on DLW patterns. a)** Optical micrographs of a hydrogel made from an aqueous solution containing 60 wt% DMAPS and 0.4 mol% (i), 0.6 mol% (ii), 1.2 mol% (iii), and 1.8 mol% (iv) crosslinker that had been patterned with AgNPs through DLW**.** Scale bars = 50 μm. **b)** Surface coverage of AgNPs (%) as a function of crosslinker concentration within the hydrogel. Maximum surface coverage of 80 % is obtained for hydrogels containing 0.6 mol% and 1.2 mol% crosslinker. **c)** Compression modulus and estimated mesh size as a function of crosslinker concentration for samples swollen in an aqueous solution containing 1 M AgNO_3_ (solid circles) and PBS (open circles). **d)** Schematic representation and optical microscopy images of patterns in as prepared samples (i), samples that have been rinsed with water (ii) and swollen in PBS (iii). Scale bars = 200 μm. **e)** Size variation with respect to the nominal design of as-prepared samples, samples that are rinsed in water, and swollen in PBS. **f)** 3D microtomography reconstructions of rectangular tracks: as-prepared (i) and after swelling in PBS (ii-iii). Scale bars = 100 μm. **g)** Surface coverage of AgNPs as a function of increasing glycerol concentration in the hydrogel. The insets display a square pattern written without glycerol (i) and with 80 vol% glycerol (ii). Scale bars = 100 μm.

The mesh size of hydrogels determines the ease for growing AgNPs to diffuse and agglomerate within the hydrogel matrix, such that we expect this parameter to also influence the surface coverage of AgNPs. To test this expectation, we quantify the surface coverage of AgNP agglomerates as a function of the hydrogel stiffness from optical micrographs. The surface coverage of AgNPs agglomerates increases if we decrease the crosslinker concentration from 1.8 mol% to 1.2 mol%, as shown in Fig. 2a and Fig. S9. Using the measured compressive modulus and rubber elasticity theory, we estimate the average mesh size of hydrogels containing 1.8 mol% crosslinker to be (3.5 ± 0.1) nm. A reduction in crosslinker concentration to 0.6 mol% increases the mesh size to (6.0 ± 0.2) nm, as shown in Fig. 2c; these values are consistent with previously reported ones [[61], [62], [63]]. We attribute the measured increase in surface coverage with decreasing crosslinker concentration to the increased mesh size that facilitates the diffusion of growing AgNPs required to form agglomerates. However, if we further decrease the crosslinker concentration to 0.4 mol%, resulting in an estimated mesh size of (7.8 ± 0.2) nm, the surface coverage decreases from (81 ± 13) % to (60 ± 13) %, as shown in Fig. 2b. We assign this behavior to the increase in mesh size of the hydrogel, which enables the diffusion of formed AgNPs beyond the region of interest, thereby effectively lowering the NP concentration in the region of interest and preventing the formation of interconnected AgNP agglomerates. These results suggest the optimum crosslinker concentration to be 0.6 mol%. Hydrogels encompassing this crosslinker concentration exhibit a Young's modulus of (33 ± 4) kPa and a strain at break of (71 ± 11) %, as shown in Fig. S7. These values are similar to those of the spinal cord and the retina in mammals [5].

A key metric for determining the quality of DLW is writing fidelity. To quantify this parameter, we measure the change in the length of the written features relative to the nominal design length immediately after the writing process is completed (as prepared), as shown in Fig. 2d(i). Remarkably, if written into optimized hydrogels containing 0.6 mol% crosslinker, features exhibit a variation as low as ± 5 % from the nominal length, as shown in Fig. 2e(i). To assess if these features maintain their shape if swollen to equilibrium, we monitor size variations during post-writing steps, including rinsing in water and subsequent swelling in PBS, as shown in Fig. 2d(ii–iii). The patterns shrink as the hydrogel is rinsed in water. For example, patterns written in hydrogels encompassing 0.4 mol% crosslinker shrink to (38 ± 2) % of their initial size. A similar degree of shrinking is measured for substrates encompassing 0.6 mol% crosslinker, as shown in Fig. 2e(ii). The shrinking is reduced to (19 ± 3) % in stiffer substrates containing 1.2 mol% crosslinker and remains at a similar level if the crosslinker concentration is increased to 1.8 mol%, as shown in Fig. 2e(ii). Notably, the size variation of the written patterns matches the swelling behavior of the hydrogel substrate, as shown by the open circles in Fig. 2e. This scaling allows to predict the final dimensions of microstructures by measuring the substrate swelling ratio, such that shrinkage-induced changes in the dimensions of the written pattern can be compensated by the initial design. Our results suggest that crosslinker concentrations between 0.6 and 1.2 mol% result in the highest AgNPs surface coverage while still offering good shape fidelity. Because we aim at using the substrates to interface with soft tissues, preference is given to softer substrates. Hence, we fix the crosslinker concentration to 0.6 mol% for the remainder of the experiments.

To enable applications in bioelectronics and tissue engineering, hydrogels must operate under physiologically relevant isotonic conditions. We thus soak the patterned substrate in PBS to quantify dimensional stability. We cannot measure any significant change in the substrate dimensions upon transfer into PBS such that the percolating network of AgNPs agglomerates remains intact and embedded within the substrate during washing and operation under physiologic conditions, as shown in Fig. 2e(iii) and confirmed with X-ray micro-computed tomography (μCT) in Fig. 2f(i-iii). Yet, μCT reveals that the conductive tracks are wrinkled at the scale of their width: this is most likely caused by the washing-induced hydrogel shrinking when transferred into deionized water.

Silver nanoparticles locally generate heat due to a strong plasmon-induced photothermal effect [64]. This localized heat evaporates water that compromises the integrity of the written structures [40,55]. To overcome this limitation, we add glycerol as a co-solvent for Ag^+^. We expect glycerol to reduce the formation of vapor bubbles during the writing process because it increases the boiling point of the solvent [65]. Glycerol also serves as a mild reducing agent, promoting the AgNPs formation [[66], [67], [68]]. To evaluate the effect of glycerol on the AgNPs formation, we measure the surface coverage as a function of glycerol concentration. In the absence of glycerol, the surface coverage is (38 ± 4) %, resulting in heterogeneous AgNP patterns, as shown in Fig. 2g. The surface coverage increases with increasing glycerol content, as visualized with the optical micrographs in Fig. 2g(i-ii) and detailed in Fig. S10. Indeed, the addition of 50 vol% glycerol yields a surface coverage of (81 ± 4) %, resulting in much more homogeneous AgNP patterns, as shown in Fig. 2g(ii). Increasing the glycerol concentration to 80 vol% only slightly increases the surface coverage to (83 ± 10) %. We obtain similar results if we replace glycerol with low molecular weight poly(ethylene glycol) (PEG), which exhibits a similar viscosity, as shown in Fig. S11 and Fig. S12.

### 2.2 DLW parameters

**Fig. 3:**
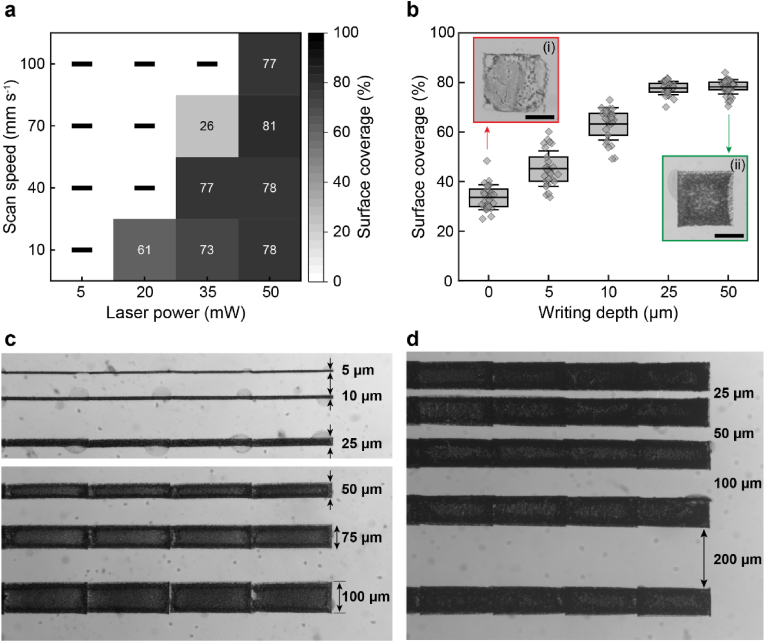
**DLW parameters and writing resolution. a)** Surface coverage of AgNPs as a function of DLW process parameters. **b)** AgNP surface coverage as a function of writing depth within the hydrogel substrate. The insets display photographs of a square pattern written at the hydrogel-glass interface (writing depth = 0 μm) (i), and 50 μm within the substrate (ii). Scale bars = 50 μm. **c)** Optical microscopy image showing the top view of AgNP tracks with widths ranging from 100 μm to 5 μm. **d)** Optical microscopy image showing tracks that are separated by 25 μm–200 μm.

Laser parameters typically influence the photoreduction-induced NP formation [40,50,51,55]. To assess if this is also the case for the system presented here, we vary power and speed of a laser with a focal spot size of 600 nm × 600 nm. If the power of this laser is 5 mW, it does not initiate photoreduction at any scan speed, as shown in the heat map in Fig. 3a. This result is consistent with the photoreduction threshold model, which suggests that a low laser power is insufficient to generate enough silver metal atoms (Ag^0^) in the focal volume to initiate nucleation and growth [40,55,69]. We start to form a significant amount of AgNPs at a laser power of 20 mW, if we scan with the slowest speed we investigated: 10 mm s^−1^. We can increase the scan speed by increasing the laser power, as summarized in Fig. 3a. Maximum surface coverage is achieved with a laser power of 50 mW. At this laser power, we can vary the scan speed between 10 mm s^−1^ and 100 mm s^−1^ without measurable difference in surface coverage, as shown in Fig. S13. Yet, the scan speed must be balanced with the density of defects that likely arise because high laser speeds risk ejecting NPs from the laser focal spot [55,56]. Based on these results, we fix the scan speed to 40 mm s^−1^ for the remainder of the experiments.

A key advantage of two-photon DLW is its ability to fabricate microstructures that are embedded within the hydrogel [50]. To evaluate the influence of the writing depth on the writing performance, we quantify the surface coverage as a function of writing depth. Patterns written at the glass–hydrogel interface, corresponding to a writing depth of 0 μm, exhibit a low surface coverage, as shown in Fig. 3b. We attribute this result to defects on the glass surface that enable AgNPs to adhere to it [56]. These AgNPs are partially detached from the hydrogel substrate upon removal of the glass slide, as shown in Fig. S14. Moreover, air bubbles that form during the writing process tend to accumulate at the glass surface, where they introduce defects into the structures while being written. These limitations can be addressed by increasing the writing depth. While air bubbles still form if the writing depth is increased beyond 10 μm, these air bubbles remain localized and their negative effect on the printing fidelity is much less severe, as shown in Movie M2. Moreover, the density of AgNPs that stick to the glass substrate decreases with increasing writing depth. As a result, the surface coverage increases with increasing writing depth, as shown in Fig. 3b. A maximum surface coverage of (78 ± 3) % is reached at a writing depth of 50 μm. These findings demonstrate the importance of optimizing laser power, scan speed, and writing depth to achieve continuous and interconnected microstructures within the substrate. As a proof-of-concept, we pattern mm-long tracks, as narrow as 5 μm and with a pitch distance down to 25 μm, as shown in Fig. 3c-d. To demonstrate the versatility of this approach, we pattern another covalently crosslinked hydrogel, poly(acrylamide) (PAAm) through DLW. Poly(acrylamide) is widely used in cell culture due to its biocompatibility, tunable stiffness, and ease of functionalization with adhesion-promoting proteins. [[70], [71], [72], [73]]. Remarkably, we obtain surface coverages up to 100 % for samples with a compression modulus between 225 kPa and 250 kPa; these values exceed those obtained for PDMAPS, as shown in Fig. 3a. Similar surface coverages as those obtained for PDMAPS can be achieved in PAAm substrates if the writing parameters and crosslinker concentrations are adjusted, as shown in Fig. S15 and Fig. S16. Note, that physically crosslinked gels with low sol-gel transition temperatures, such as gelatin, cannot be used as substrates because AgNP-induced photothermal effects locally melt the substrate during the writing process, compromising writing fidelity, as shown in Movie S3.

Supplementary data related to this article can be found online at https://doi.org/10.1016/j.mtbio.2025.102140

The following are the Supplementary data related to this article.

video 3 video 3

video 4 video 4

### 2.3 Electronic properties of patterned microstructures

**Fig. 4:**
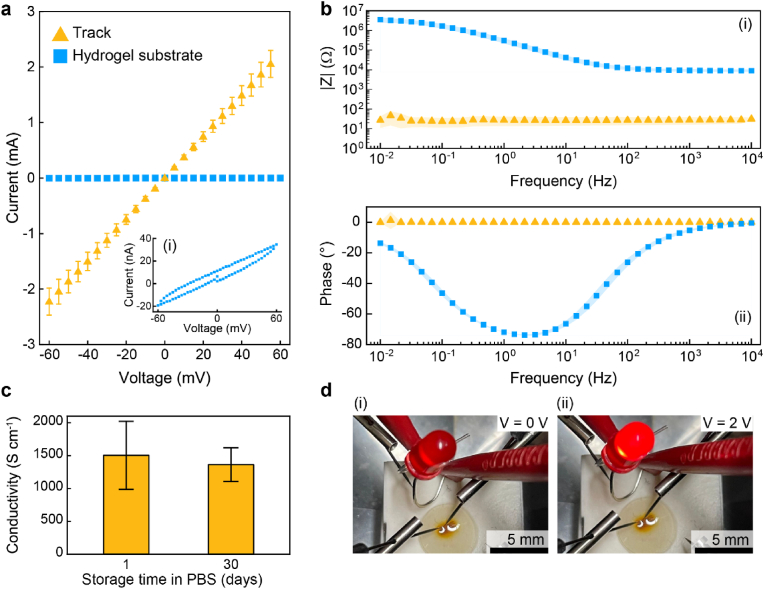
**Electronic properties of silver microstructures. a)** Current-voltage (I–V) plot of the silver microstructures (yellow triangles) and the hydrogel substrate (blue squares). The inset provides a magnified view of the I-V characteristics of the hydrogel substrate (i). Tungsten tips mounted on micromanipulators used to establish contact with the silver tracks embedded within the hydrogel substrate. **b)** Two-electrode impedance spectroscopy of the microstructures (yellow triangles) and the hydrogel substrate (blue squares). The microstructure exhibits frequency-independent impedance in the modulus (i) and phase (ii), confirming its purely electronic conductivity. In contrast, the hydrogel substrate soaked in PBS displays capacitive behavior at frequencies below 10^2^ Hz, due to ionic conductivity. **c)** Conductivity of the microstructures as a function of storage time in PBS. The conductivity of the tracks is (1505 ± 518) S cm^−1^, and shows no significant change after 30 days of storage in PBS. **d)** Photograph showing the illumination of a red LED when the circuit is closed through the silver microstructure and voltage is increased from 0 V (i) to 2 V (ii). (For interpretation of the references to colour in this figure legend, the reader is referred to the Web version of this article.)

Our results suggest that AgNPs form a percolating network. To assess if they impart electronic conductivity to PDMAPS, we perform current-voltage (I-V) sweeps on patterned tracks located within this hydrogel swollen in PBS. To ensure a good electrical connection between the probe tips and tracks, we fabricate tracks with laser parameters that yield the highest surface coverage (∼80 %). Tracks prepared with lower surface coverage fail to ensure good electrical contact, as shown in Fig. S17. The tracks exhibit a linear I-V relationship, as shown in Fig. 4a and Fig. S18. Tracks with typical dimensions of 2000 μm in length, 100 μm in width, and 10 μm in thickness exhibit a resistance of (27 ± 3) Ω, reaching a current of 2 mA if we apply a voltage of 60 mV. When the same voltage is applied to the bare hydrogel substrate, currents only reach 40 nA, two orders of magnitude lower than in the patterned devices, as shown by the I-V inset in Fig. 4a(i).

Owing to their hydrophilicity and high swelling capacity, hydrogels are good ion conductors. The ionic conductivity of PDMAPS increases from 7 mS cm^−1^ to 11 mS cm^−1^ with decreasing crosslinker concentration, as reported in Fig. S19. These values are similar to those previously reported for zwitterionic hydrogels [74,75]. To decouple ionic from electronic conductivity, we conduct 2-electrode electrochemical impedance spectroscopy (EIS) on the microstructures and the hydrogel substrate. The impedance modulus and phase of the microstructure demonstrate a frequency-independent behavior, as shown in Fig. 4b(i-ii). This is expected for a metallic conductor with purely electronic conductivity [20,24,76]. The mean value of |Z| of (28 ± 4) Ω throughout the tested frequency range is similar to the resistance value of (27 ± 3) Ω extracted from DC measurement. Conversely, the hydrogel substrate swollen in PBS-glycerol exhibits a frequency-dependent behavior, arising from the interfacial double layer capacitance and the geometric capacitance of the hydrogel, as shown in the blue curve of Fig. 4b(i).

Patternable hydrogels might be integrated into bioelectronic devices. Yet, this application requires the electronic properties of the microstructure to remain stable over time. To assess long-term stability of our samples, we measure the conductivity of the structures after 1 and 30 days of storage at room temperature in PBS. A conductivity of (1505 ± 518) S cm^−1^ is recorded after one day. Remarkably, the conductivity remains nearly unchanged after 30 days of storage, (1362 ± 257) S cm^−1^, as shown in Fig. 4c. These values are similar to those reported for conductive hydrogel systems that have been functionalized with metallic silver nanofillers [7,17,50,77,78]. These finding suggests that the formed AgNPs agglomerates remain stable for at least 30 days even if immersed in salt-containing aqueous solutions. Small Ag nanoparticles have been reported to oxidize over time [[79], [80], [81]]. Our results suggest that the agglomeration of AgNPs occurring during the DLW process renders them less prone to oxidative dissolution, thereby increasing their stability in aqueous solutions. Remarkably, the storage modulus of the substrate is not significantly affected by the presence of AgNP fillers, as shown in Fig. S20. This is in stark contrast to conventional hydrogel nanocomposites prepared by introducing pre-formed fillers, whose rheological properties have to be traded off with the filler concentration [[82], [83], [84]]. This result suggests that the hydrogel substrate can be tailored to meet the mechanical requirements of bioelectronic and tissue engineering applications, being minimally affected by the electronically conductive patterned structures. Note that the resistance changes only by 17 % from (29 ± 2) Ω to (24 ± 1) Ω if samples are 3 times strained to ∼50 %, as shown in Fig. S21. These results indicate that our substrate can sustain repeated strains with values similar to those experienced by brain and spinal cord during normal postural movements [5].

To demonstrate the potential of the electronically conductive tracks located within hydrogels for applications that require small currents, we use these microstructures to close an electrical circuit that successfully powers a light-emitting diode (LED), as shown in Fig. 4d.

**Fig. 5:**
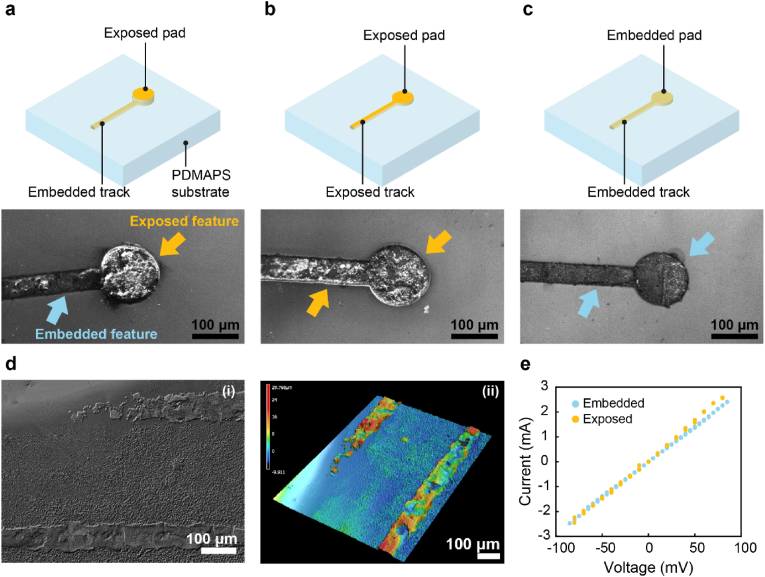
**DLW of microstructures with exposed and embedded features**. Schematic views of the design with exposed (yellow arrow) and embedded features (blue arrow), and corresponding optical microscopy images. **a)** Exposed pad and embedded track. **b)** Exposed pad and track. **c)** Embedded pad and track. **d)** Surface topology and 3D reconstruction of tracks patterned on the hydrogel surface. **e)** I-V curve of exposed (yellow) and embedded track (blue). (For interpretation of the references to colour in this figure legend, the reader is referred to the Web version of this article.)

Bioelectronic applications often desire encapsulation of the conductive features to protect the electrode [3]. To demonstrate the versatility of our method in positioning written structures within the hydrogel matrix, we fabricate microstructures containing exposed and hydrogel-encapsulated three-dimensional features: tracks are written at a depth of 10 μm such that they are embedded whereas the pads are exposed at the surface to facilitate contact with external electronic equipment, as illustrated in Fig. 5a–c. To demonstrate the low resistance of exposed patterns, we fabricate 40 μm thick tracks starting at a writing depth of 40 μm, so that the top layer of the track is exposed at the hydrogel surface whereas the remainder of the track is embedded, as shown in Fig. 5d. Exposed tracks show linear I-V relationship with a resistance value of ∼32 Ω, similar to that of embedded ones, as shown in Fig. 5e and Fig. S22. The low track resistance is beneficial for recording applications, as it minimizes the thermal noise introduced by the metal interconnects, as shown in Fig. S23. We envision combining embedded and exposed conductive features to enable fully hydrogel-based devices that can be more easily interfaced with external electronic equipment.

**Fig. 6:**
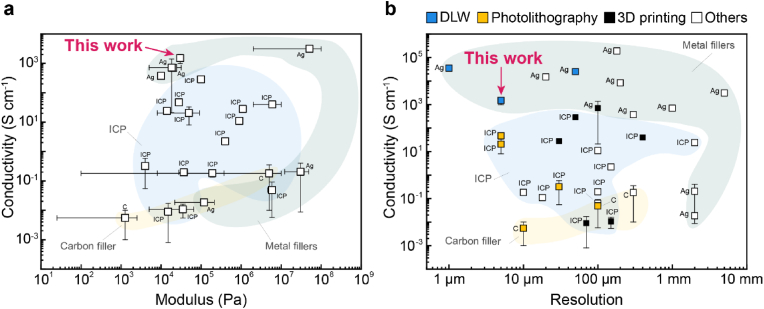
**Patternable and electronically conductive hydrogels**. Ashby plots comparing the conductivity versus **a)** modulus and **b)** resolution of conductive hydrogels. The comparison includes silver-based composites (green area), intrinsically conductive polymer-based composites (ICP, blue area), and carbon-based composite (yellow area). Data points are from references reported in Fig. S24 and Table S1. (For interpretation of the references to colour in this figure legend, the reader is referred to the Web version of this article.)

Our findings suggest that our system reaches electrical performance similar to those of other Ag-based hydrogel composites without compromising the mechanical and rheological properties, as summarized in Fig. 6a. DLW achieves a writing resolution similar to that of photolithographic patterning of PEDOT:PSS samples, as shown in Fig. 6b. However, because structures are written voxel-by-voxel, the writing process is time-consuming; it takes ∼15 min to write a 2 mm-long track. Despite this limitation, the voxel-by-voxel writing approach enables printing 3D structures, as we demonstrate by printing exposed pads connected to embedded tracks within a single processing step, which sets this technique apart from standard photolithography techniques. The maximum dimensions of these structures in the xy plane are limited by the travel range of the substrate holder stage; in our case, the maximum area that can be written within a single processing step is ∼8.5 mm^2^. The maximum height of features that can be written is ∼190 μm and is limited by the objective working distance. With further development focused on multi-scale patterning to support electrical interconnects using standardized techniques, and the implementation of strategies to encapsulate conductive patterns with dielectric layers, we foresee this approach to hold potential for fabricating electronically conductive, patternable hydrogels for soft bioelectronics and *in vitro* tissue engineering.

## 3 Conclusion

We introduce a method to pattern electronically conductive 3D silver microstructures within hydrogel substrates. Patterned conductive tracks can be fabricated with typical dimensions ranging from 100 μm to several millimeters, and exhibit a conductivity of (1505 ± 518) S cm^−1^. This is achieved through two-photon direct laser writing-induced photoreduction of silver ions within a hydrogel substrate possessing a Young's modulus as low as (33 ± 4) kPa. By varying the substrate composition and DLW parameters, we achieve a writing fidelity down to ±5 %. With our proposed approach, different hydrogel formulations, metal ions, and device geometries can be explored. With further work focused on the introduction of electrical insulation of the patterned tracks, spatially selective recording and stimulation could be achieved. Moreover, by assessing the biocompatibility of the samples and developing designs to facilitate the connection of the conductive hydrogel to external equipment, this technique has the potential to advance the integration of hydrogel-embedded metallic features into engineered devices for soft bioelectronics and *in vitro* tissue engineering.

## 4 Methods

### 4.1 Materials

[2-(Methacryloyloxy)ethyl]dimethyl-(3-sulfopropyl)ammonium hydroxide (DMAPS) (Sigma-Aldrich, 537284), acrylamide (AM) (40 wt% solution, ThermoScientific, J62480.AP), acrylic acid (AA) (Sigma-Aldrich, 147230), 2-Acrylamido-2-methyl-1-propanesulfonic acid (AMPS) (Sigma-Aldrich, 282731), glycerol (Thermofisher, 7530.1), N,N′-Methylenebisacrylamide (MBAA) (Carl Roth, 7867.1), 2-Hydroxy-2-methylpropiophenone (PI) 97 % (Sigma-Aldrich, 405655), ammonium persulfate (APS) (Sigma-Aldrich, 248614), silver nitrate (Carl Roth, 9370.4), phosphate buffer saline 1x (PBS) (Gibco), poly(ethylene glycol) (PEG200, Mw ∼200 g mol^−1^)(Carl Roth, 2631.1) All materials are used as received.

### 4.2 Preparation of PDMAPS hydrogel substrates

To prepare PDMAPS hydrogels, an aqueous solution containing 60 wt% DMAPS, varying concentrations of MBAA as crosslinker (0.4 mol%, 0.6 mol%, 1.2 mol%, and 1.8 mol%, with respect to the monomer), and 5 μL mL^−1^ (with respect to the water volume) PI was prepared. The solution was degassed under nitrogen flow, then casted in a PTFE mold (thickness = 2 mm) and crosslinked for 5 min under UV light (UWAVE, UV-365-100, 366 nm, 50 mW cm^−2^). The resulting crosslinked hydrogel was demolded and rinsed for 48 h in deionized water to remove unreacted reagents. After rinsing, hydrogel disks were cut using an 8 mm puncher. Hydrogel disks were dried for 4 h in an oven at 70 °C (VWR Dry-Line). Dried hydrogels were soaked for at least 48 h in an aqueous solution containing 1 M AgNO_3,_ 50 vol% glycerol (unless otherwise specified), and 10 μL mL^−1^ PI. Samples were stored at 4 °C covered from light until further use.

### 4.3 Preparation of PAAm hydrogel substrates

To prepare PAAm hydrogels, an aqueous solution containing 40 wt% acrylamide, varying concentrations of MBAA used as a crosslinker (0.6 mol%, 1.0 mol%, and 1.2 mol%, with respect to the monomer), and 1 w/v % APS used as a thermal initiator was prepared. The solutions were cast in a PTFE mold (thickness = 2 mm) and crosslinked for 1 h at 70 °C. The crosslinked hydrogels were demolded and rinsed for 48 h in deionized water to remove unreacted reagents. Rinsed hydrogel disks were cut using an 8 mm diameter puncher. Hydrogel disks were dried for 4 h in an oven at 70 °C (VWR Dry-Line). Dried hydrogels were soaked for at least 48 h in an aqueous solution containing 1 M AgNO_3,_ 50 vol% glycerol (unless otherwise specified), and 10 μL mL^−1^ PI. Samples were stored at 4 °C, covered from light until further use.

### 4.4 Direct laser writing of metallic microstructures within PDMAPS hydrogel substrates

A two-photon direct laser writer (DLW) (Photonics Professional GT+, Nanoscribe) was used to fabricate metallic microstructures within the hydrogel substrates. The DLW operates with a femtosecond pulsed laser, 780 nm, 80 MHz repetition rate, using a 25× objective lens (NA = 0.8), in oil immersion mode. Hydrogel substrates were blotted with filter paper to remove excess solution and then placed on a borosilicate glass slide (diameter: 30 mm, thickness: 170 μm). Unless otherwise specified, the hatching distance was 0.7 μm, the slicing distance was 1 μm, the laser power was 50 mW (corresponding to 100 % laser power in NanoWrite software), and the scan speed was 40 mm s^−1^. After writing, hydrogel substrates containing metallic features were rinsed in de-ionized water (ρ = 20 MΩ cm, 23 °C) for at least 48 h to remove unreacted species. Rinsing bath was replaced every 24 h. After rinsing, samples were stored at room temperature in PBS or in de-ionized water.

### 4.5 Swelling rate measurement

To measure the swelling rate of hydrogel substrates with varying crosslinker concentrations, samples were dried for 4 h in an oven at 70 °C, and then soaked in an aqueous solution containing 50 vol% glycerol and 1 M AgNO_3_. The swelling rate (*SR*) was calculated as:

$$SR\;\left(\%\right)=\frac{m_{t}-m_{0}}{m_{0}}\times 100$$

where *m*_*t*_ is the weight of the hydrogel substrate at time *t*, and *m*_*0*_ is the weight of the dried sample. Measured data are reported as (mean ± standard deviation), and are representative of at least three independent measurements.

### 4.6 Measurement of hydrogel substrate swelling ratio in various swelling media

To evaluate the influence of successive swelling media on hydrogel substrate size, we measured the diameter swelling ratio after sequential immersion and swelling to equilibrium in three solutions. First, hydrogels were immersed in an aqueous solution containing 1 M AgNO_3_ and 50 vol% glycerol. Next, the same hydrogels were transferred to water and swollen to equilibrium. Finally, they were swollen in PBS. The diameter swelling ratio (*SR*_*d*_) was calculated for each swelling medium as follows:

$$SR_{d}\;\left(\%\right)=\frac{d_{s}-d_{0}}{d_{0}}\times 100$$

where *d*_*s*_ is the diameter of the hydrogel substrate, and *d*_*0*_ is the initial diameter of the hydrogel substrate. Measured data are reported as (mean ± standard deviation), and are representative of at least three independent measurements.

### 4.7 Surface coverage quantification

To assess surface coverage under different writing conditions, we patterned squares with a nominal size of (100 x 100) μm (width x length) and 1 μm thickness. The laser power was fixed at 50 mW and the scan speed at 40 mm s^−1^, unless otherwise specified. Patterns were imaged with an inverted microscope (Nikon Eclipse TS100) with 20x lens equipped with a digital camera (Sony, XCG-CG240C). To quantify the surface coverage, images were thresholded via Otsu's method using a custom-made macro on ImageJ. Surface coverage was defined as:

$$Surface\;coverage\;\left(\%\;\right)=\frac{A_{c}-A_{ROI}}{A_{ROI}}\times 100$$

where *A_c_* is the area of the square covered by metal nanoparticles, and *A*_*ROI*_ is the area of region of interest (ROI). For each pattern, we define ROI as the smallest square that completely encloses the patterned area. A graphical explanation of surface coverage is reported in Fig. S25. Measured data are reported as (mean ± standard deviation), and are representative of at least 45 independent measurements. All box plots are reported with 25 %–75 % percentile, mean value line, and ±standard deviation. Statistical significance is tested via Tukey's multiple comparison test on OriginPro (2021b).

### 4.8 Mechanical characterization of hydrogel substrates

Tensile tests were performed on dog-bone shaped samples (cross section area = 16 mm^2^) with a commercial machine (ZwickiLine, 50 N load cell, Zwick Roell) with a constant velocity of 100 mm min^−1^. The Young's modulus was calculated as the slope of the initial linear region, from 5 % to 15 % strain. Compression tests were performed on cylindrical samples (thickness = 8 mm, diameter = 8 mm) with a commercial machine (ZwickiLine, 5 kN load cell, Zwick Roell), compressed at a constant velocity of 5 mm min^−1^ until fracture or 80 % strain was reached. The compression modulus was calculated as the slope of the initial linear region, from 5 % to 15 % strain. All samples for tensile and compressive tests were soaked at equilibrium in de-ionized water or in a water solution containing 1 M AgNO_3_ and 50 vol% glycerol. Measured data are reported as (mean ± standard deviation), and are representative of at least 3 independent measurements.

### 4.9 Rheological characterization of hydrogel substrates

PDMAPS substrates for rheological measurement (thickness = 2 mm, diameter = 8 mm) were prepared from an aqueous solution containing 60 wt% DMAPS and varying concentrations of crosslinker. All samples were swollen to equilibrium in PBS before the measurement. Rheological measurements were performed on a DHR-3 TA Instrument with a 20 mm diameter parallel plate steel geometry. Samples were blotted with filter paper to remove excess water from the sample surface before the measurement was initiated. All measurements were performed at 37 °C, with 0.1 N axial force applied to the sample. Amplitude sweeps were performed at 1 Hz for a strain range of 0.1 %–300 %. Measured data are reported as (mean ± standard deviation), and are representative of at least 3 independent measurements.

### 4.10 Hydrogel mesh size calculation

Hydrogel mesh size was estimated using a previously reported model [62]. The mesh size, *ξ*, was estimated as:

$$\xi =\sqrt[{3}]{\frac{k_{B}T}{E_{comp}}}$$

where *k*_*B*_ is the Boltzmann constant, *T* is the temperature, and *E*_*comp*_ is the compressive modulus of the hydrogel substrate as measured by compressive test. Measured data are reported as (mean ± standard deviation), and are representative of at least 3 independent measurements.

### 4.11 Electrical characterizations of silver microstructures

Electrical characterization was performed on tracks swollen to equilibrium in 1x PBS containing 50 vol% glycerol, to reduce drying during the measurement. Electrical resistance of the hydrogel matrix and the silver microstructures was measured using a two-probe station (MMR Technologies) connected to a picoammeter (Keithley 6487) and an electrometer (Keithley 6517A). Silver microstructures were connected using tungsten tips mounted on micromanipulators. Voltage was scanned from −60 mV to +60 mV at 8 mV/s. Electrical resistance was extracted from the linear fit (Linear Fit, Origin 2021b) of current-voltage (I-V) plot. Conductivity (*σ*) of microstructures was computed as:

$$\sigma =R\frac{L}{t\times W}$$

where *R* is the resistance extracted from the linear fit of the I-V plot, and *L, W,* and *t* are the length, width, and thickness of the track, respectively. *L* and *W* are *L* = (2156 ± 25) μm, *W* = (105 ± 12) μm, as measured from optical images. The thickness of the tracks was *t* = 10 μm, as measured from micro-tomography scans. Electrochemical Impedance Spectroscopy (EIS) of silver microstructures and hydrogel substrate was performed in a two-electrode configuration with a potentiostat (Gamry Interface 1010B). The applied voltage was 10 mV and the frequency range was 10 mHz to 10 kHz. Measured data are reported as (mean ± standard deviation), and are representative of at least 3 independent measurements.

### 4.12 Ionic conductivity of PDMAPS hydrogel substrates

PDMAPS hydrogels were prepared following the same protocol used for fabricating PDMAPS substrates for DLW. For ionic conductivity measurements, samples swollen to equilibrium in 1x PBS were punched into discs with an approximate thickness of 2 mm and a diameter of 10 mm. Each disc was placed between two gold-coated glass slides, separated by a rubber spacer. Two-electrode EIS was performed at 10 mV over a frequency range of 100 mHz to 10 kHz. Ionic resistance was determined by fitting the data to the equivalent circuit model shown in Fig. S19.

### 4.13 Optical profilometry

Optical profilometry of tracks exposed on the hydrogel surface was performed with Keyence VK‐X200 laser microscope. Images were acquired in Differential Interference Contrast (DIC) mode. 3D rendering of the surface profile was plotted with Keyence VK Analyzer software.

### 4.14 SEM imaging

Scanning electron microscopy (SEM) of AgNPs formed via DLW was performed on a Zeiss Gemini 300, with a working distance of 6 mm, 3 kV operating voltage, using a secondary electron detector or backscattered electrons. Hydrogel samples were prepared by flash freezing in liquid nitrogen, followed by freeze drying, and coating with 10 nm of evaporated carbon.

### 4.15 μCT imaging and 3D reconstruction of silver microstructures within hydrogel substrates

X-Ray microcomputed tomography (μCT) was performed on an Ultratom micro tomography system (RX-SOLUTIONS). The sample was scanned at a voxel resolution of 900 nm, with a voltage of 80 kV and a current of 140 μA. ImageJ was used for 3D reconstruction and visualization.

## CRediT authorship contribution statement

**Lorenzo Lucherini:** Writing – review & editing, Writing – original draft, Validation, Methodology, Formal analysis, Data curation, Conceptualization. **Veronica Navello:** Data curation. **Outman Akouissi:** Writing – review & editing, Conceptualization. **Stéphanie P. Lacour:** Writing – review & editing, Supervision, Conceptualization. **Esther Amstad:** Writing – review & editing, Writing – original draft, Supervision, Project administration, Investigation, Funding acquisition, Conceptualization.

## Additional information

Supplementary information is available in the online version of the paper. Correspondence and requests for materials should be addressed to E. A.

## Declaration of generative AI and AI-assisted technologies in the writing process

During the preparation of this work the author(s) used ChatGPT-4o in order to improve the readability of the manuscript. After using this tool/service, the author(s) reviewed and edited the content as needed and take(s) full responsibility for the content of the published article.

## Declaration of competing interest

The authors declare the following financial interests/personal relationships which may be considered as potential competing interests: Esther Amstad reports financial support was provided by 10.13039/501100001711Swiss National Science Foundation. If there are other authors, they declare that they have no known competing financial interests or personal relationships that could have appeared to influence the work reported in this paper.

## Data Availability

Data will be made available on request.
