# Supplementary material for: Direct laser writing of electronically conductive microstructures within soft hydrogel substrates
Source: Mater Today Bio. 2025 Jul 30;34:102140. doi: 10.1016/j.mtbio.2025.102140 (PMC12345326; doi:10.1016/j.mtbio.2025.102140)
Supplement: Multimedia component 1 [file mmc1.docx]

Supporting information

**Direct Laser Writing of Electronically Conductive Microstructures within Soft Hydrogel Substrates**

Lorenzo Lucherini,^1^ Veronica Navello,^1^ Outman Akouissi,^2,3^ Stéphanie P. Lacour,^2^ Esther Amstad ^1,*^

^1^ Soft Materials Laboratory, Institute of Materials, École Polytechnique Fédérale de Lausanne, Lausanne, Switzerland.
^2^ Laboratory for Soft Bioelectronic Interfaces, Neuro-X institute, École Polytechnique Fédérale de Lausanne (EPFL), Geneva, Switzerland.
^3^ Bertarelli Foundation chair in translational neuroengineering, Neuro-X institute, École Polytechnique Fédérale de Lausanne (EPFL), Geneva, Switzerland.

*E-mail: [esther.amstad@epfl.ch](mailto:esther.amstad@epfl.ch)

**Movie M1.** Direct laser writing (DLW) of a silver microstructure. The hydrogel substrate is PDMAPS made from an aqueous solution containing 60 wt% monomer and 0.6 mol% crosslinker. The microstructure is written at a writing depth of 100 µm. The laser power is 25 mW and the scan speed is 5 mm s^-1^. The playback speed is increased 4-times.

**Movie M2.** DLW of (100 x 100) µm square patterns at varying writing depths within the hydrogel substrate. The writing depth is defined according to the following reference system. The hydrogel substrate is PDMAPS made from a solution containing 60 wt% monomer and 0.6 mol% crosslinker. The laser power is 50 mW and the scan speed is 40 mm s^-1^.

**
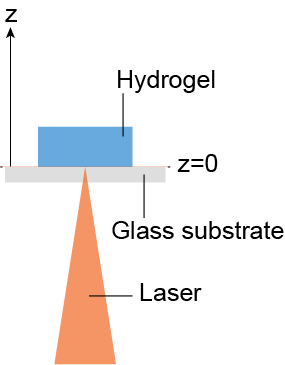
**

**Movie M3.** DLW of (100 x 100) µm square pattern on a gelatin hydrogel substrate prepared with 15 wt% gelatin. Writing depth is 50 µm. The laser power is 50 mW and the scan speed is 40 mm s^-1^.


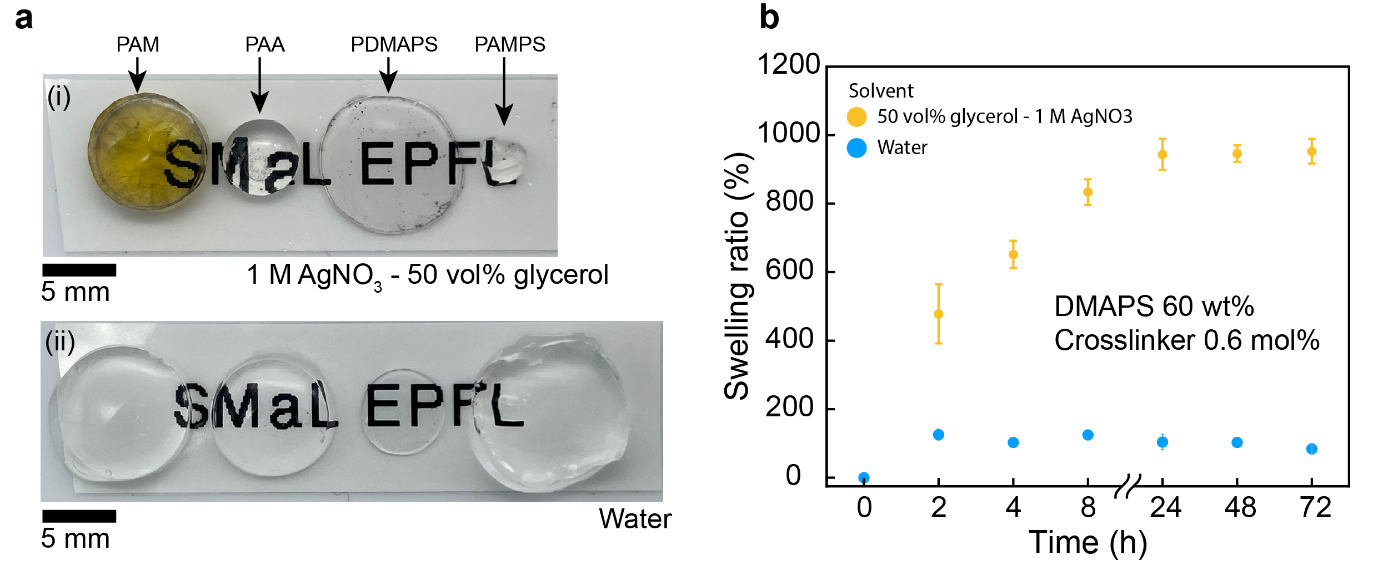


**Supplementary Figure S1. Hydrogel substrate design for DLW. a)** Photograph of different covalently-crosslinked hydrogel substrates tested for direct laser writing (DLW): polyacrylamide (PAM), polyacrylic acid (PAA), poly-[2-(Methacryloyloxy)ethyl]dimethyl-(3-sulfopropyl)ammonium hydroxide (PDMAPS), and poly-(2-Acrylamido-2-methylpropane sulfonic acid) (PAMPS). Photographs show the substrate at swelling equilibrium in an aqueous solution containing 1 M AgNO_3_ and 50 vol% glycerol (i) and water (ii). **b)** Mass swelling ratio of a poly-DMAPS (PDMAPS) hydrogel prepared from a solution containing 60 wt% DMAPS and 0.6 mol% crosslinker soaked in an aqueous solution containing 1 M AgNO_3_ and 50 vol% glycerol (yellow circles) and in water (blue circles).

**
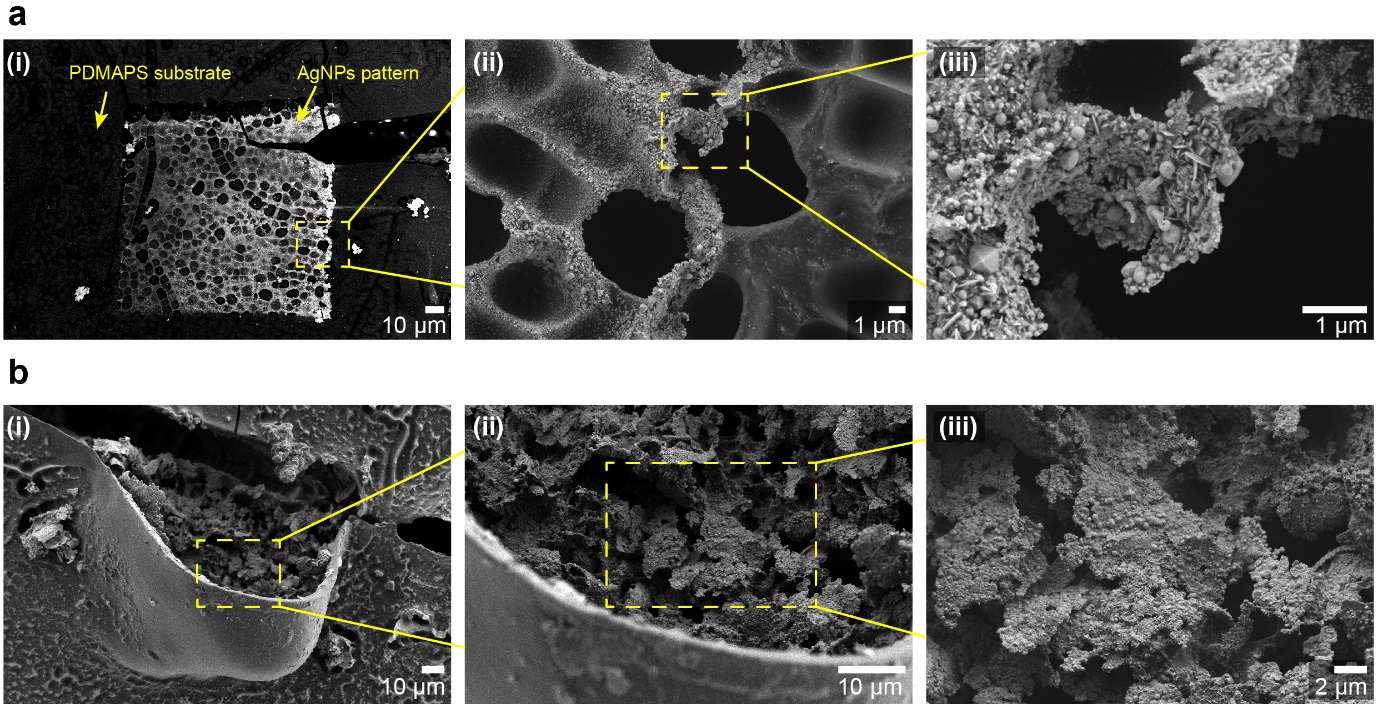
** **Supplementary Figure S2. Scanning electron microscopy (SEM) image of AgNPs formed via DLW.** AgNPs formed **a)** on the substrate surface and **b)** within the hydrogel substrate. The substrate material is PDMAPS made from solutions encompassing 60 wt% monomer and 0.6 mol% crosslinker. Laser power is 50 mW and scan speed is 40 mm s^-1^.


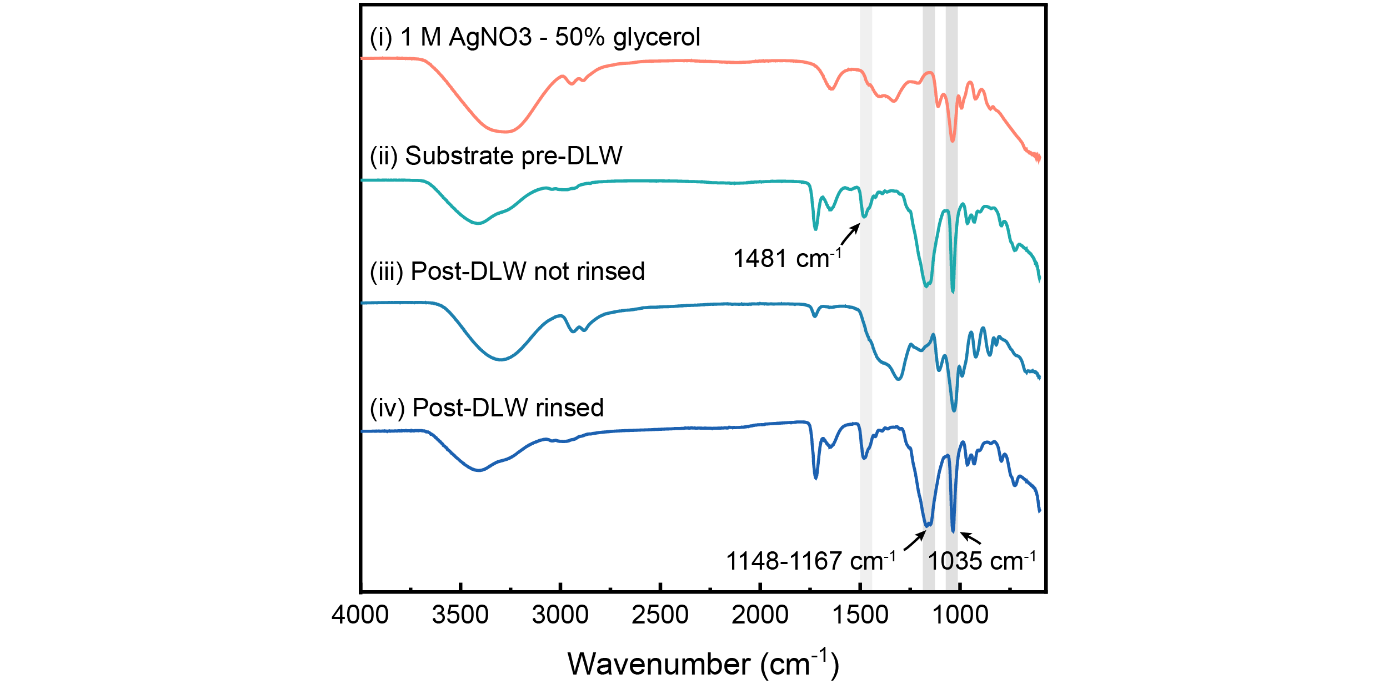


**Supplementary Figure S3. Fourier transform infrared spectroscopy (FTIR) of PDMAPS before and after the DLW process.** FTIR spectra of (i) water containing 1 M AgNO_3_ and 50 vol% glycerol, (ii) PDMAPS before swelling in a metal precursor solution for DLW, (iii) after DLW and before rinsing, (iv) and after rinsing**.** The peak at 1481 cm^-1^ is assigned to the quaternary ammonium group [1] and the peaks between 1148-1167 cm^-1^ and at 1035 cm^-1^ to the S=O vibration peaks of PDMAPS [2,3]**.**

**
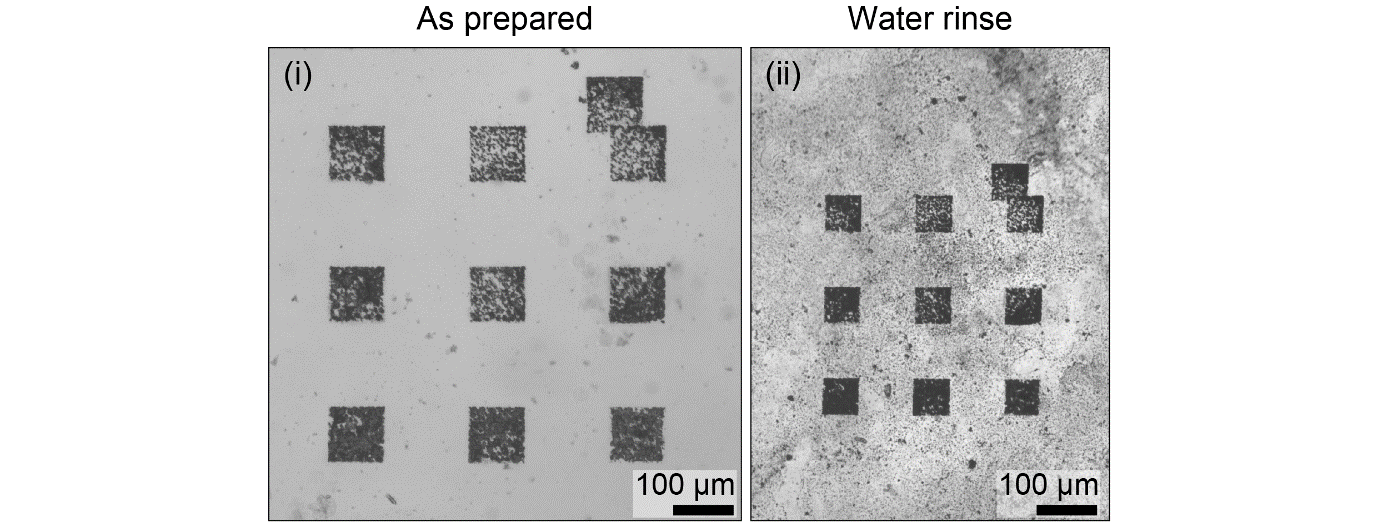
**

**Supplementary Figure S4. Size reduction of patterned features on PDMAPS substrate upon water rinse.** Optical microscopy images of squared patterns after the DLW process is completed (as prepared) (i), and after rinsing in de-ionized water (ii). The substrate was prepared from an aqueous solution containing 60 wt% monomer and 0.6 mol% crosslinker.


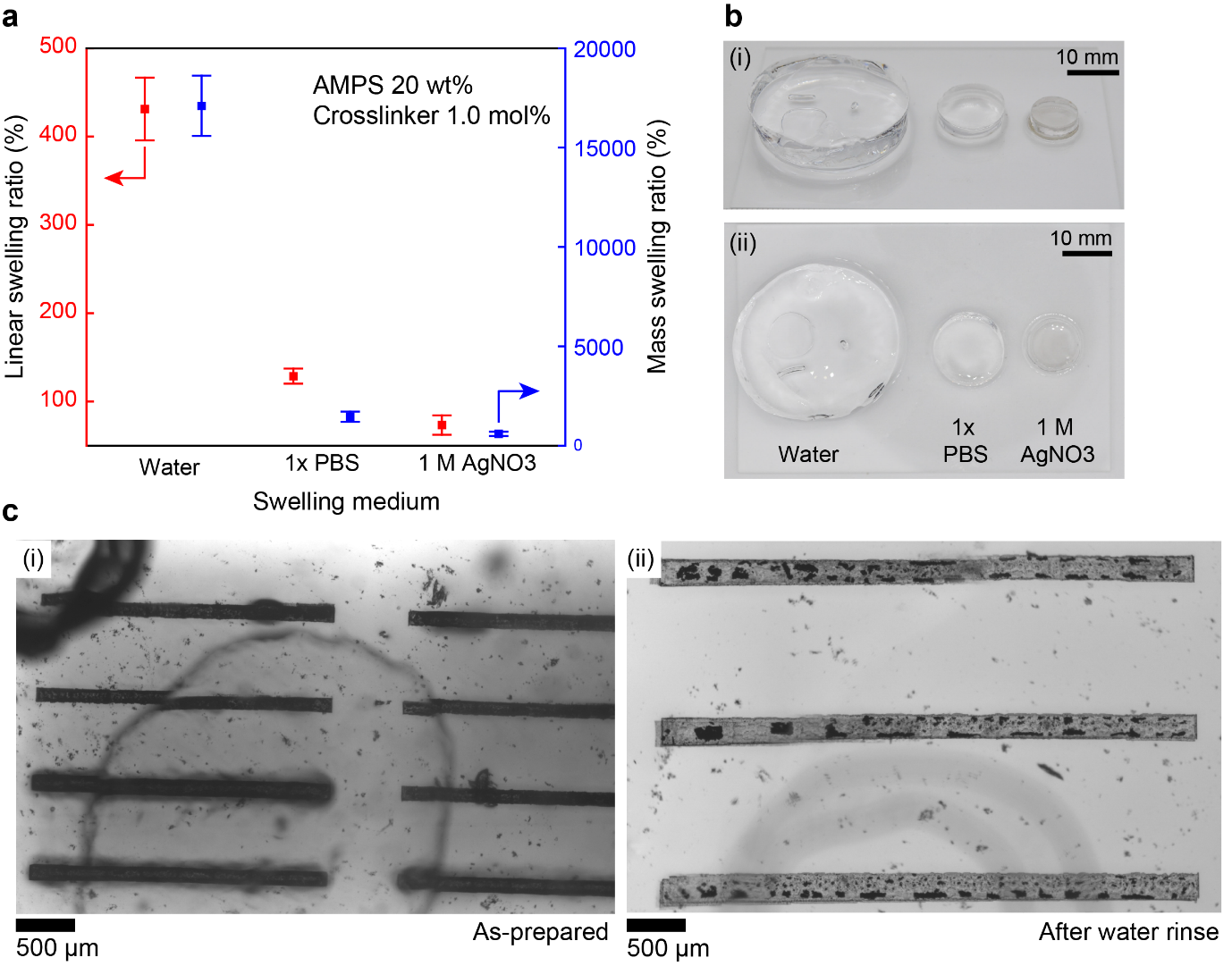


**Supplementary Figure S5. Washing-induced damages of patterned tracks in PAMPS hydrogels. a)** Linear (red) and mass (blue) swelling ratio of PAMPS. **b)** Photograph of PAMPS swollen to equilibrium in water, 1x PBS, and aqueous solution containing 50 vol% glycerol and 1 M AgNO_3_**. c)** Optical microscopy images showing microstructures within PAMPS (i) as-prepared and (ii) after rinsing with water. The swelling ratio of PAMPS in water is higher than in silver ion-containing aqueous solutions, causing the rupture of the percolating network of AgNPs upon water rinse.

**Supplementary Figure S6**. **Swelling ratio of PDMAPS in PBS**. Mass swelling ratio of PDMAS containing different crosslinker concentrations in PBS as a function of time.


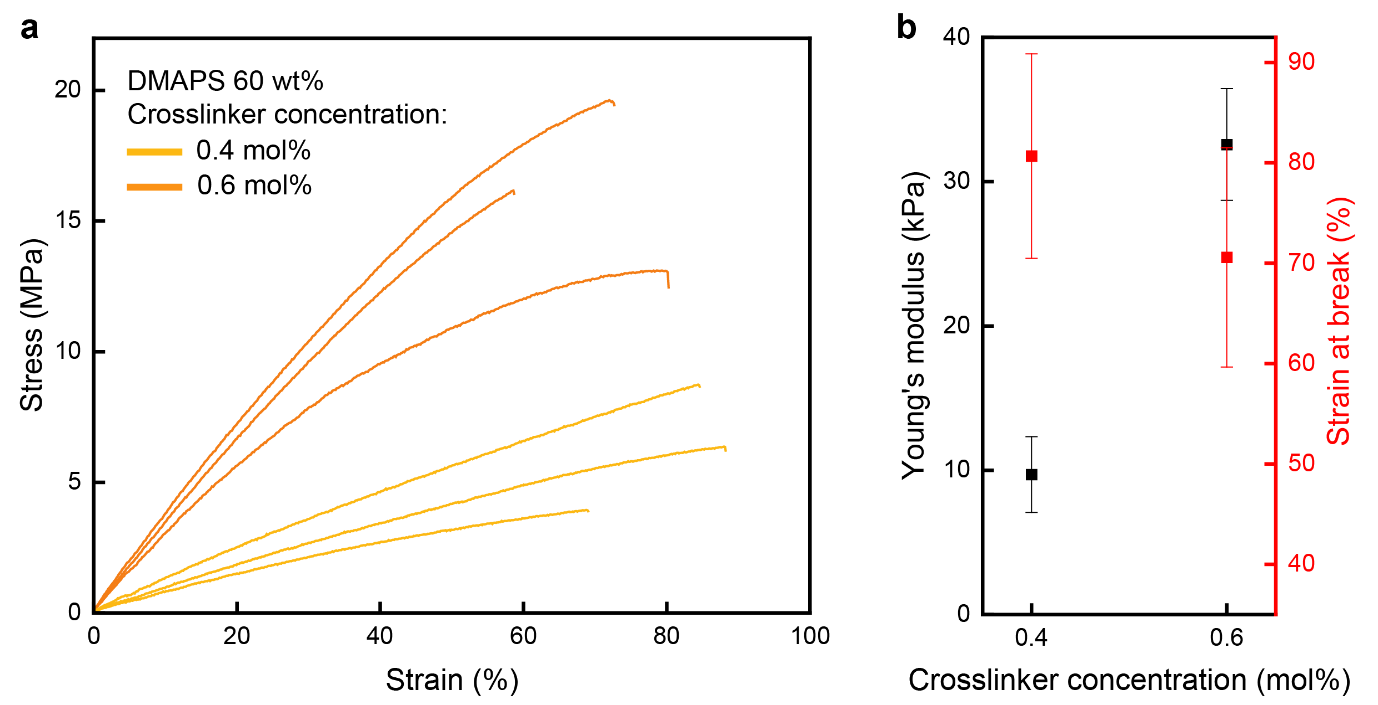


**Supplementary Figure S7. Mechanical properties of PDMAPS substrates.** **a)** Tensile stress-strain curves for PDMAPS made from an aqueous solution containing 60 wt% monomer and varying crosslinker concentrations. **b)** Young’s modulus and strain at break of PDMAPS.

**
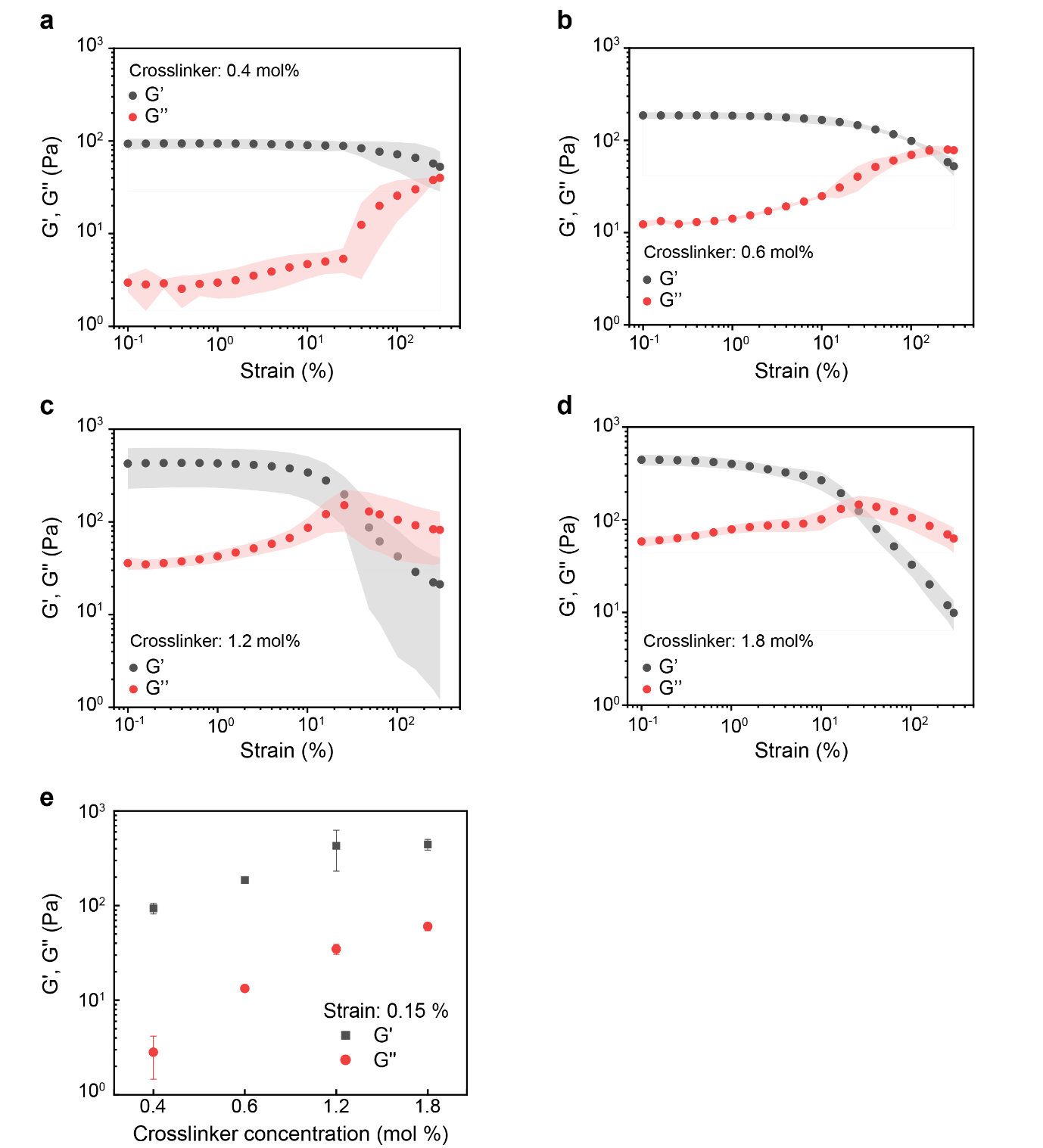
**

**Supplementary Figure S8.** Rheological characterization of PDMAPS. Hydrogels were prepared from aqueous solutions containing 60 wt% DMAPS and **a)** 0.4 mol%; **b)** 0.6 mol%; **c)** 1.2 mol%, **d)** 1.8 mol% crosslinker. **e)** Storage and loss modulus at 0.15% strain as a function of crosslinker concentration. All substrates were swollen to equilibrium in PBS before the measurements. The error band represents the standard deviation for each data point.

**
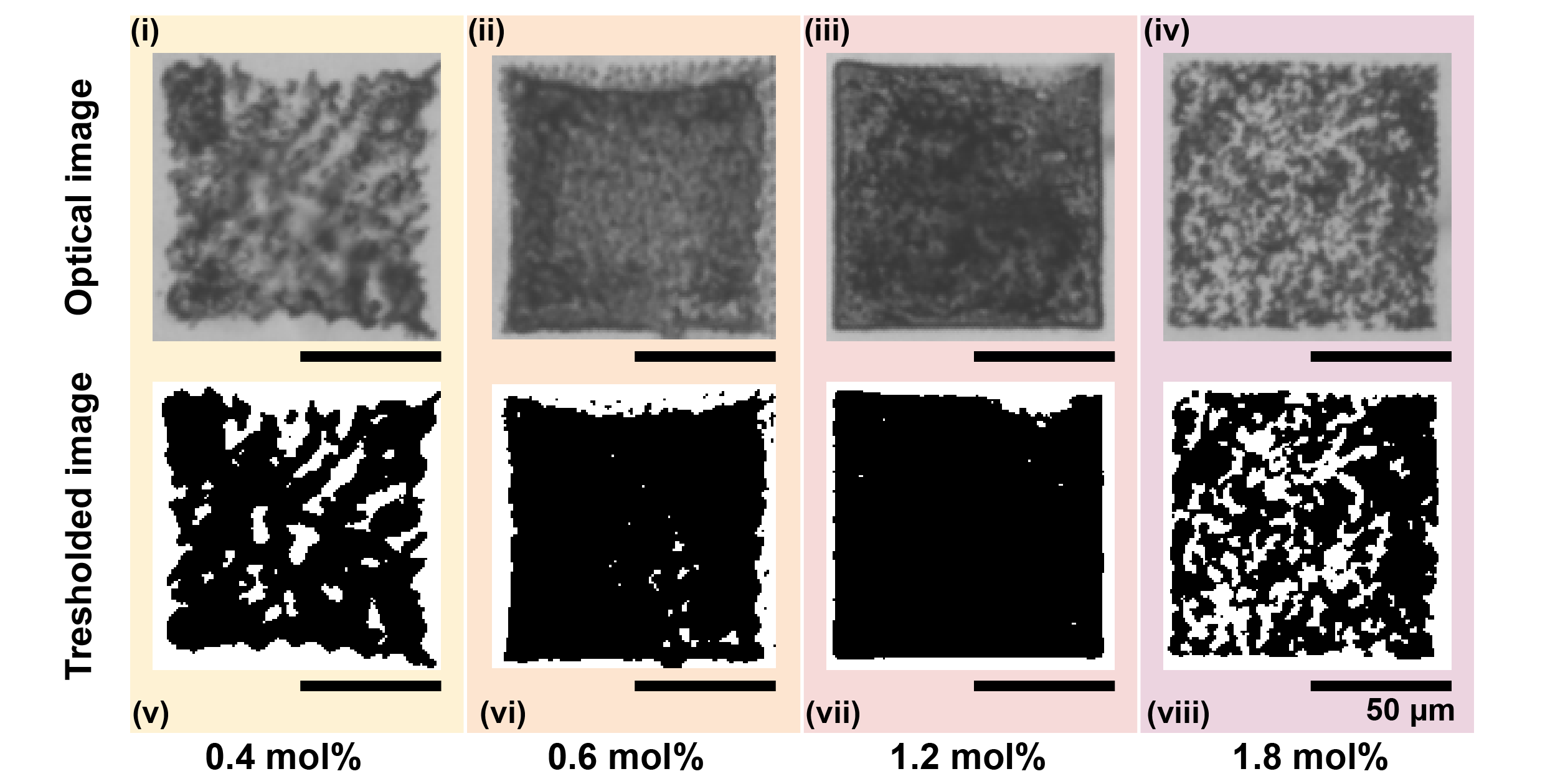
**

**Supplementary Figure S9. Quantification of AgNP formation via DLW.** Optical images of square patterns composed of AgNPs for increasing crosslinker concentrations (i, ii, iii, iv) and respective thresholded images (v, vi, vii, viii).


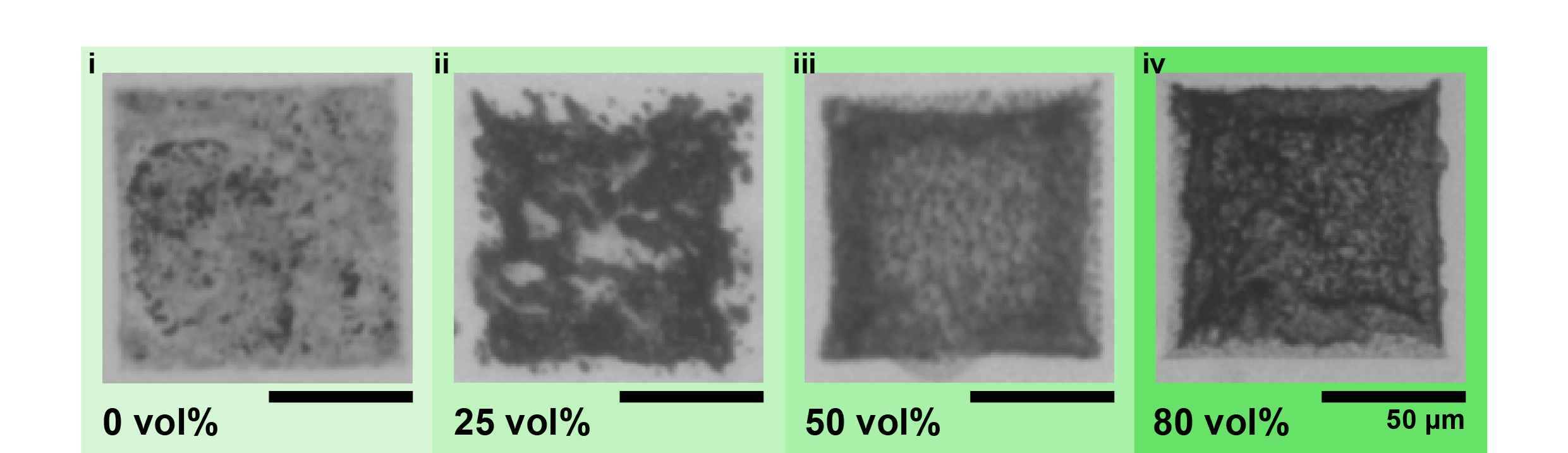


**Supplementary Figure S10. Optical micrographs of patterns written in hydrogels containing increasing glycerol concentrations.** PDMAPS made from an aqueous solution containing 60 wt% monomer and 0.6 mol% crosslinker is used as a substrate. Laser power is 50 mW and scan speed is 40 mm s^-1^. Samples are swollen in a water containing 1 M AgNO_3_ and 0 vol% (i), 25 vol% (ii), 50 vol % (iii), and 80 vol% (iv) glycerol.


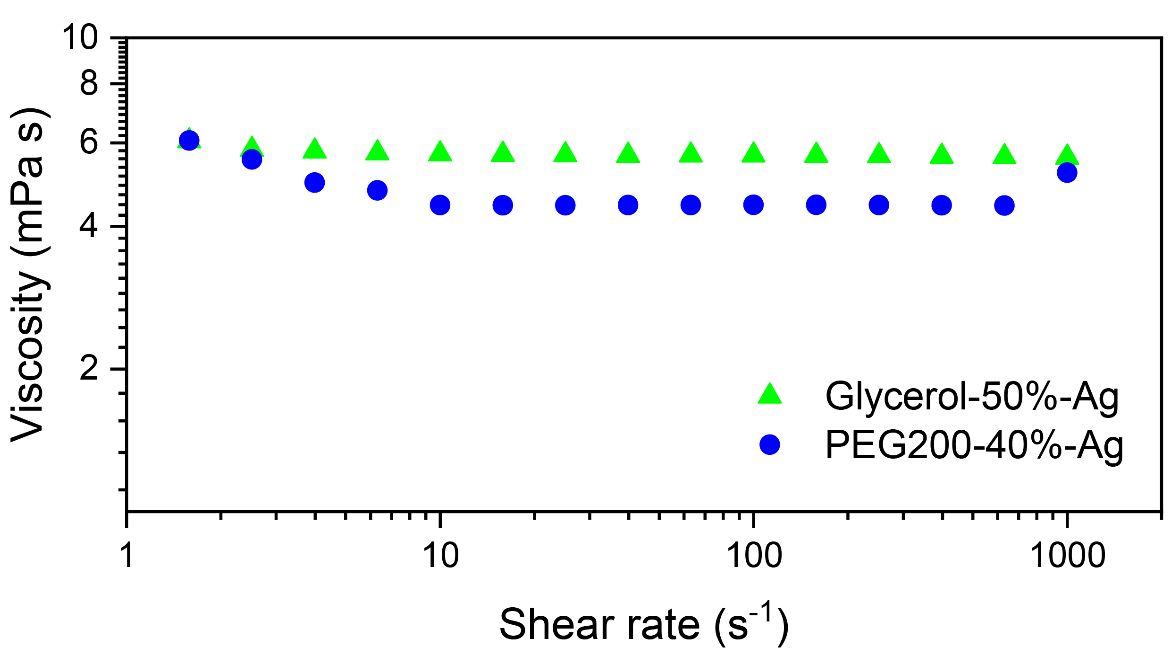


**Supplementary Figure S11. Viscosity measurements.** Viscosity measurements of water containing: 1 M AgNO_3_ and 50 vol% glycerol (green triangle), 1 M AgNO_3_ and 40 vol% poly(ethylene glycol) (PEG200) (blue dot).


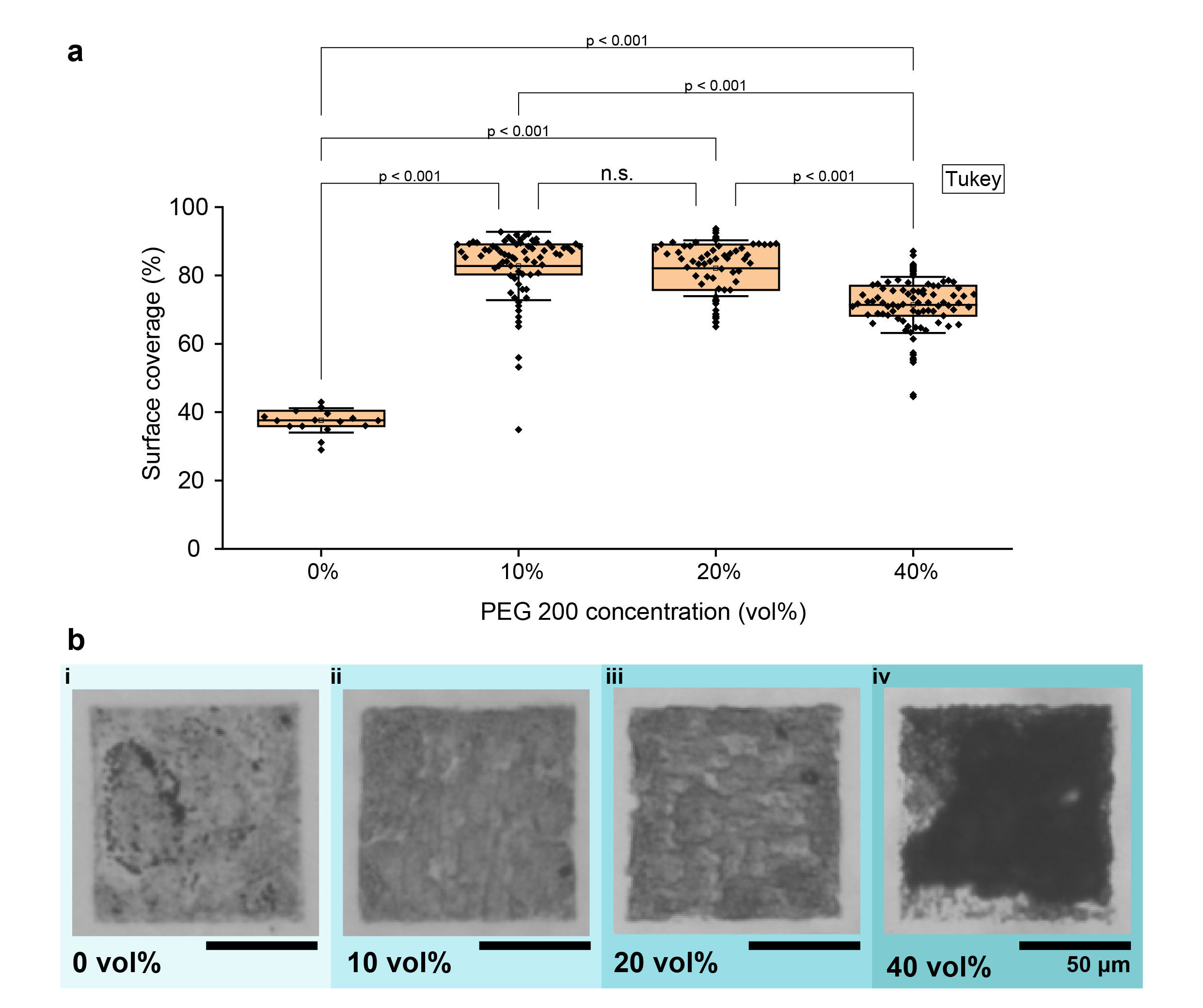


**Supplementary Figure S12. Surface coverage of patterns with increasing poly(ethylene glycol) (PEG 200) concentrations**. **a)** Surface coverage as a function of increasing PEG 200 concentration. **b)** Optical micrographs of patterns written with increasing PEG 200 concentration. made from an aqueous solution containing 60 wt% monomer and 0.6 mol% crosslinker is used as a substrate. Laser power is 50 mW and scan speed is 40 mm s^-1^. Samples are swollen in a water solution containing 1 M AgNO_3_ and 0 vol% (i), 10 vol% (ii), 20 vol % (iii), and 40 vol% (iv) PEG 200 Da.


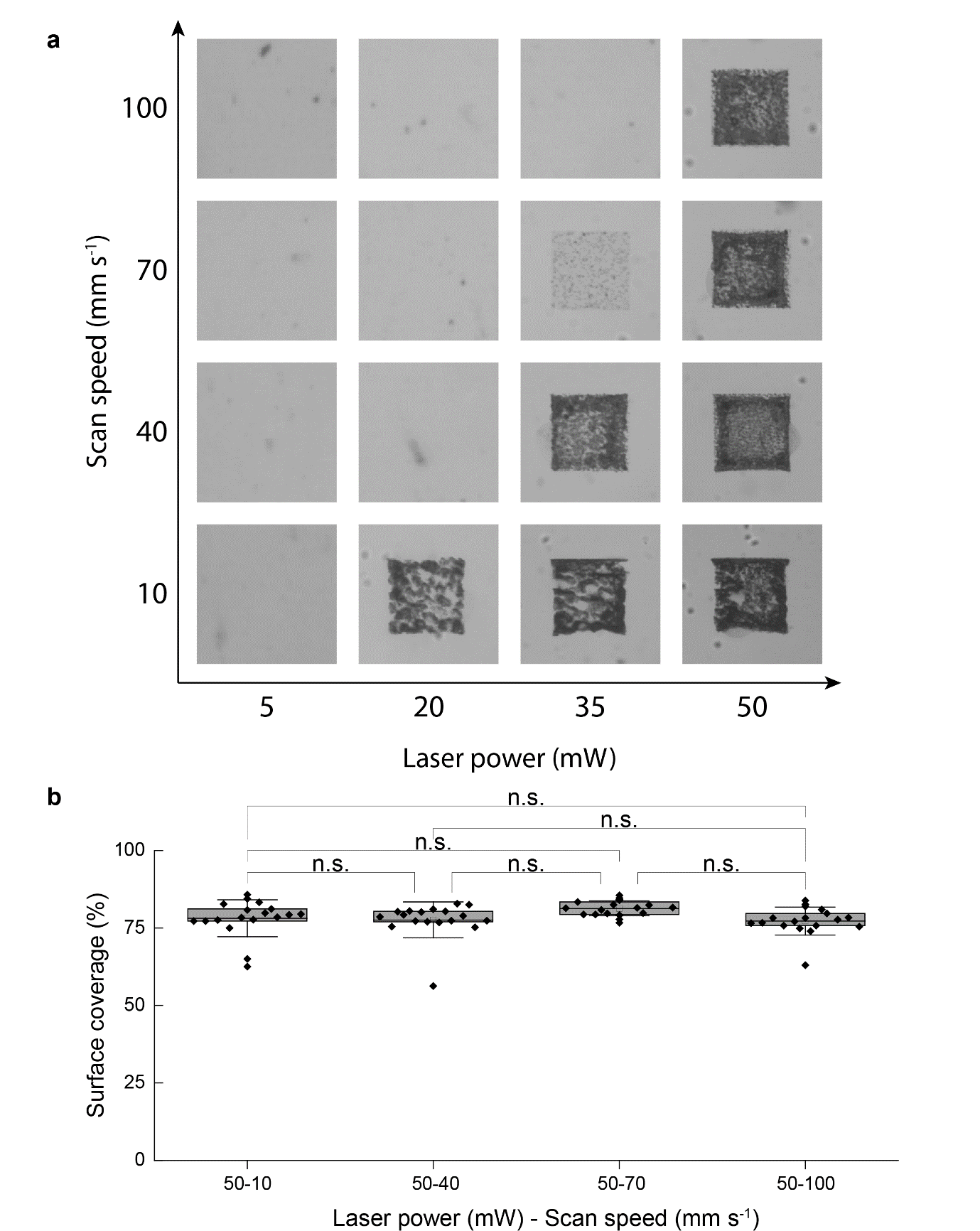


**Supplementary Figure S13. Dose matrix for DLW process parameters. a)** Optical images of tested combinations of laser power and scan speed**. b)** Surface coverage of patterns written at 50 mW laser power and increasing scan speed.


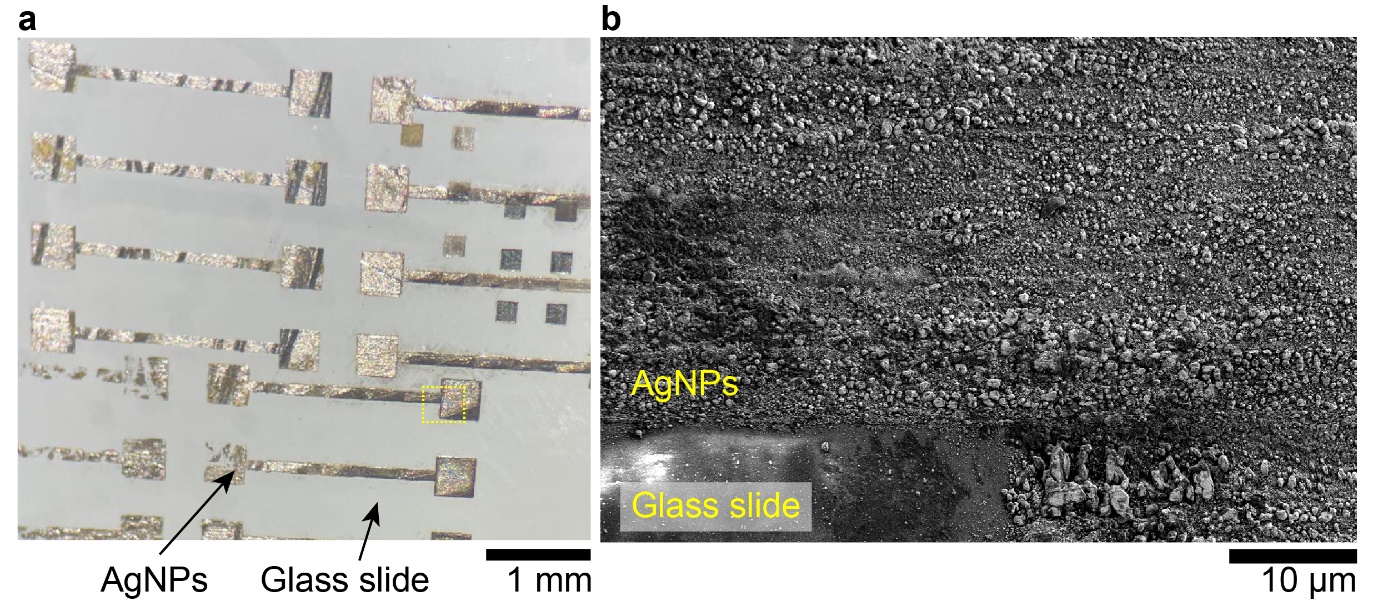


**Supplementary Figure S14. Writing at hydrogel-glass interface. a)** Optical microscopy image of AgNPs formed on a glass slide with a writing depth = 0 µm (glass-hydrogel interface). **b)** SEM of AgNPs that adhered on the glass after writing such that they were ripped off the hydrogel substrate.


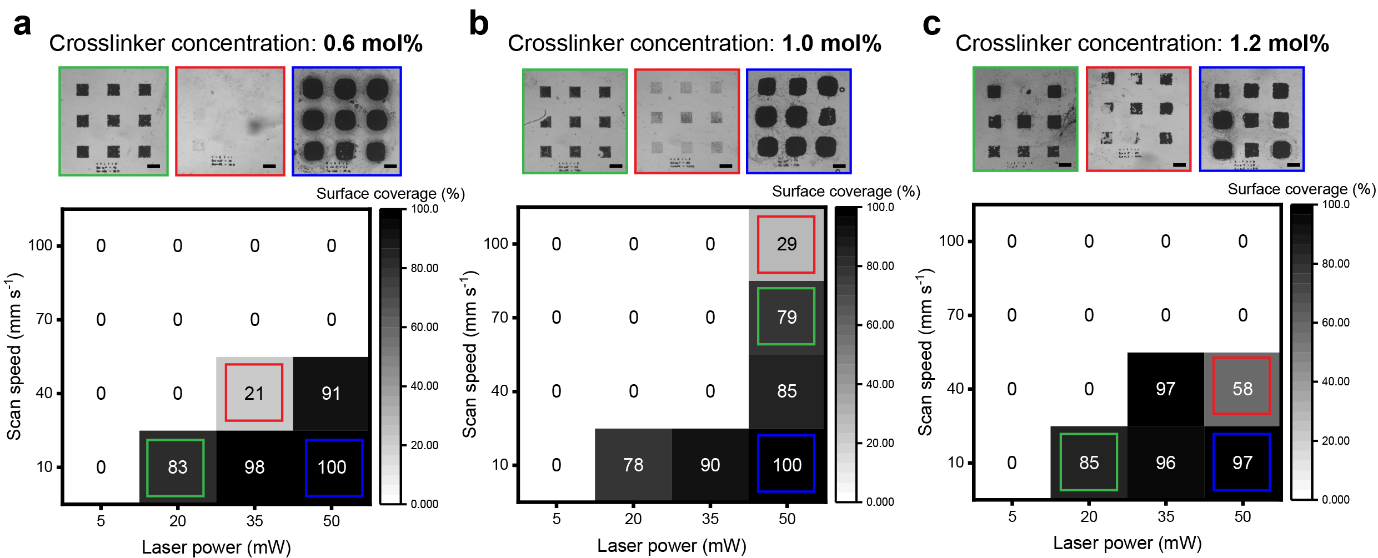


**Supplementary Figure S15. DLW on polyacrylamide (PAAm).** Optical microscopy images and dose matrix for DLW on PAAm made from aqueous solutions containing 40 wt% monomer and **a)** 0.6 mol%, **b)** 1.0 mol%**, c)** 1.2 mol% crosslinker. Colored outlines of the optical images show the corresponding point on the dose matrix. Each color indicates the combination of DLW parameters that yields: surface coverage similar to that obtained on PDMAPS substrates (green), minimum surface coverage observed (red), maximum surface coverage observed (blue). Scale bars = 100 µm.

**Supplementary Figure S16. Compressive modulus of PAAm.** Compressive modulus of PAAm used for DLW as a function of the crosslinker concentration. Samples have been swollen to equilibrium in PBS before the measurement. Results are reported as mean ± standard deviation and are representative of at least 3 independent measurements.


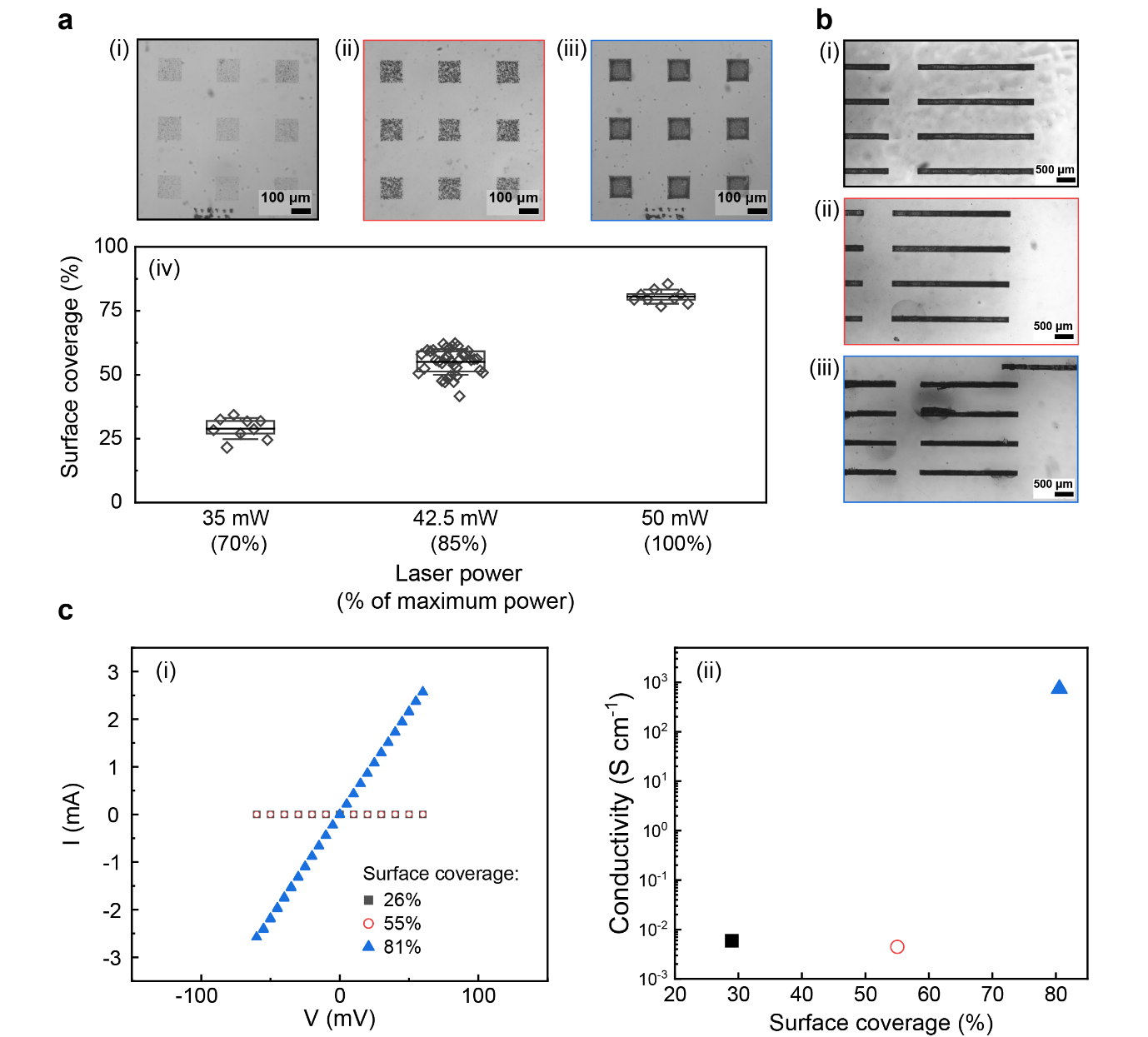


**Supplementary Figure S17. Electrical behavior of patterned tracks written with increasing surface coverage. a)** Optical images of patterns used for surface coverage measurement at fixed scan speed of 70 mm s^-1^ and 35 mW (i), 42.5 mW (ii), and 50 mW (iii) laser power. Quantification of surface coverage as a function of the laser power (iv). **b)** Tracks with thickness ~10 µm patterned at fixed scan speed of 70 mm s^-1^ and increasing laser power: 35 mW (i), 42.5 mW (ii), and 50 mW (iii). **c)** I-V plot of tracks patterned with increasing surface coverage: 26% (grey square), 55% (red open circle), 81% (blue triangle) (i); conductivity as a function of increasing surface coverage (ii).


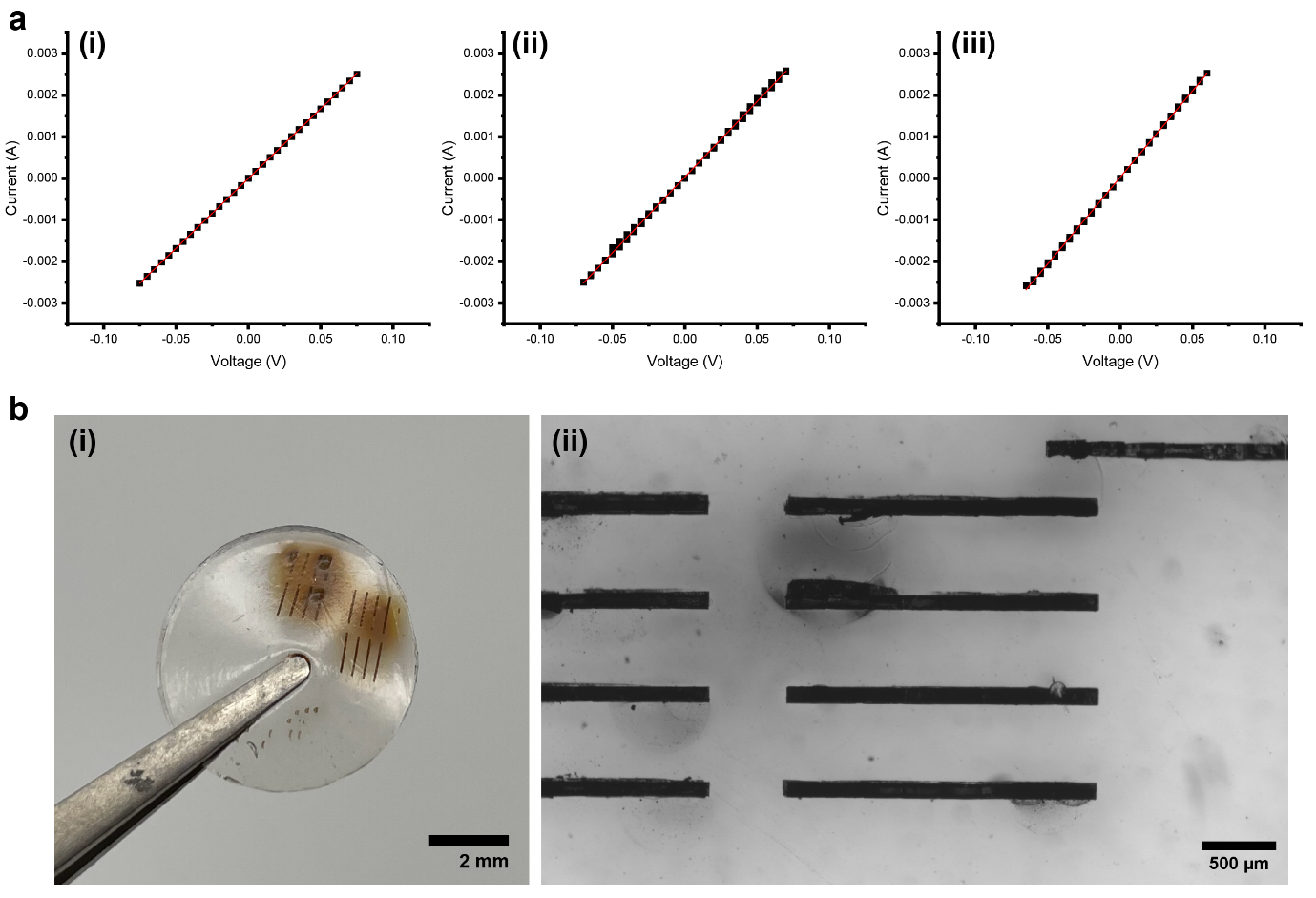


**Supplementary Figure S18. Electrical characterization of silver microstructures.** **a)** Linear fit of current-voltage (I-V) measurements on rectangular tracks. Data points are reported as black squares and linear fit as continuous red line. Fit parameters: (slope = 0.03358, R^2^ = 0.99999) (i), (slope = 0.03639, R^2^ = 0.99914) (ii), (slope = 0.04159, R^2^ = 0.99957) (iii) **b)** photograph of tracks used for I-V measurement (i) and optical microscopy image showing a close up of the track geometry (ii).


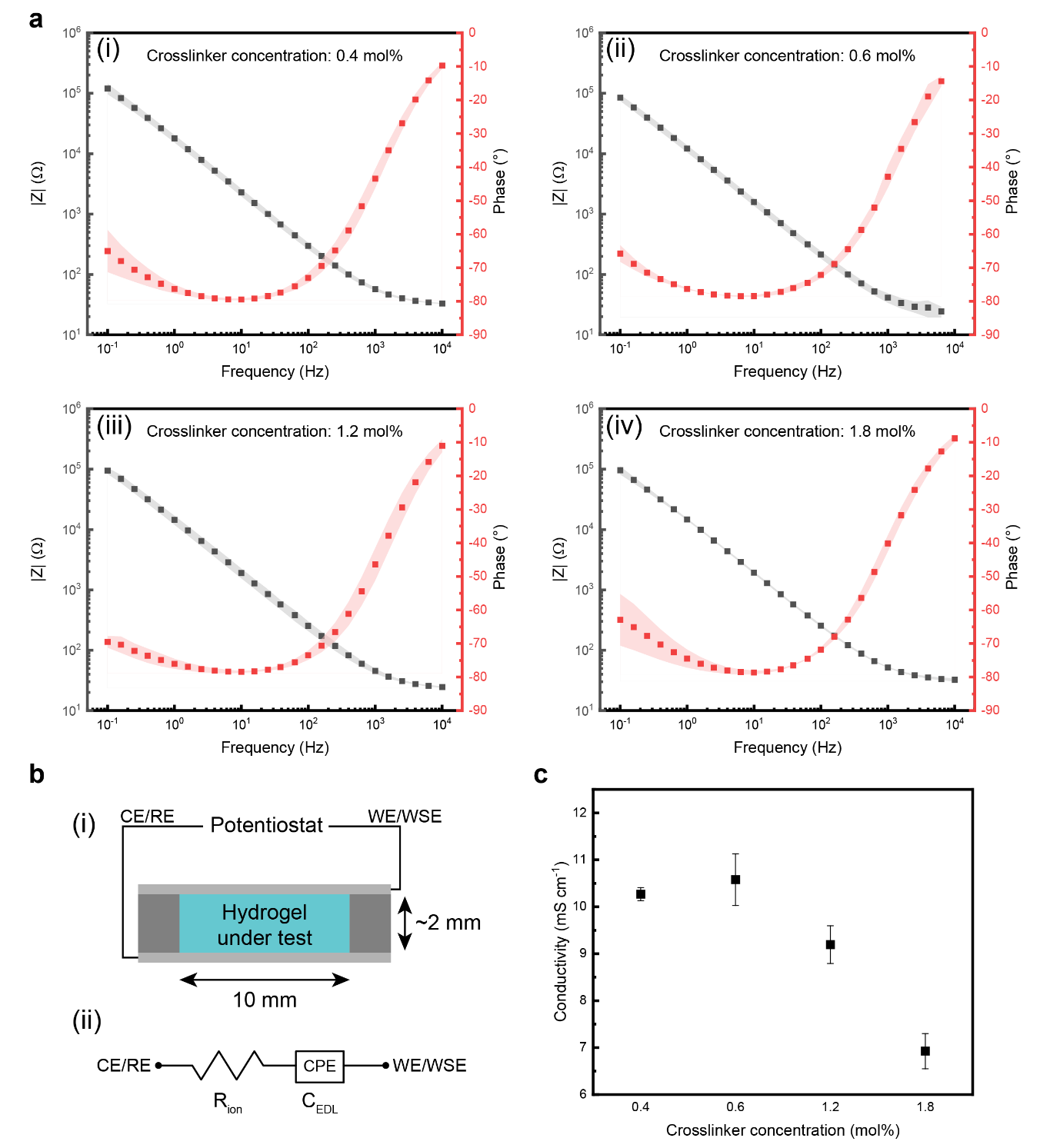


**Supplementary Figure S19. Electrical characterization of PDMAPS.** **a)** Two-electrode EIS performed on substrates made from aqueous solutions containing 60 wt% DMAPS and (i) 0.4 mol %, (ii) 0.6 mol%, (iii) 1.2 mol% and (iv) and 1.8 mol% crosslinker. **b)** Schematic illustration of the two-electrode setup used for EIS measurement (i) with the equivalent circuit used to fit EIS data (ii). CE: counter electrode; RE: reference electrode; WE: working electrode; WSE: working sense electrode. *R_ion_* models the ionic resistance, *C_EDL_* represents the constant phase element (CPE) corresponding to non-ideal double layer capacitance. **c)** Conductivity extracted from *R_ion_* as a function of the crosslinker concentration.


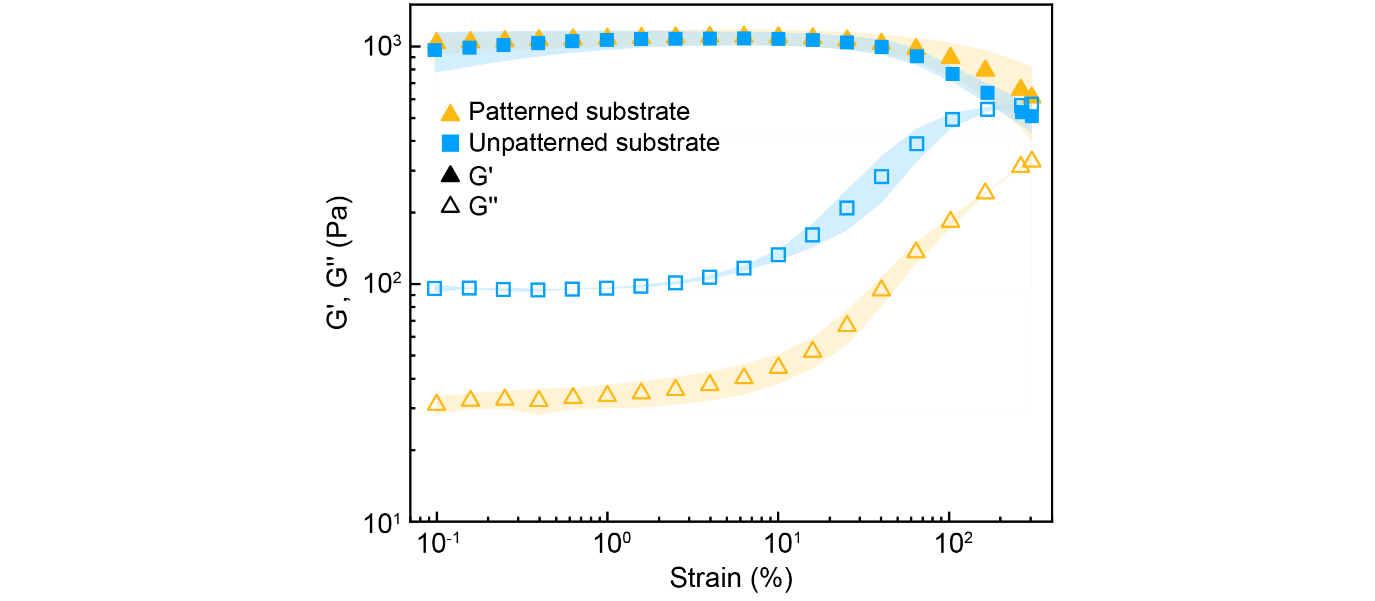


**Figure S20. Rheology of PDMAPS before and after DLW**. Storage modulus (G’, filled symbol) and loss modulus (G’’, empty symbol) of PDMAPS without patterns (square) and with patterned tracks (triangle). Substrates have been made from a solution containing 60 wt% DMAPS and 0.6 mol% crosslinker. Samples were swollen to equilibrium in PBS containing 50 vol% glycerol.


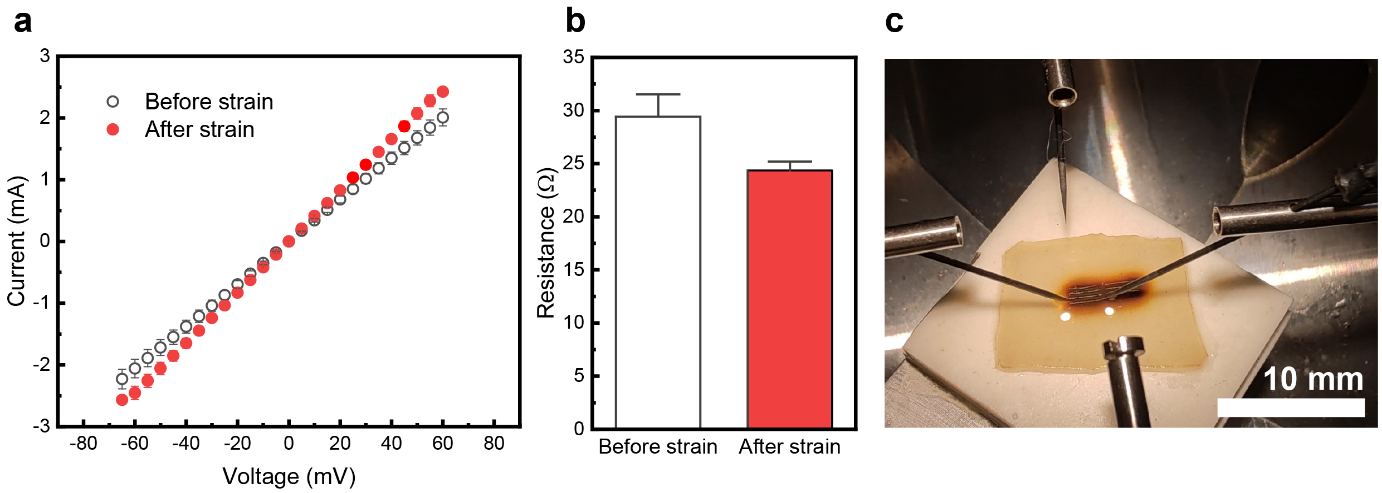


**Supplementary Figure S21. Resistance of patterned tracks before and after straining. a)** I-V curves before (empty circle) and after (full circle) applying ~50 % strain to the sample**. b)** Resistance before and after the application of 50% strain on the substrate. **c)** Two-probe setup used to measure the resistance after stretching the sample. Data are reported as mean and standard deviation of results performed on at least 3 independent measurements.


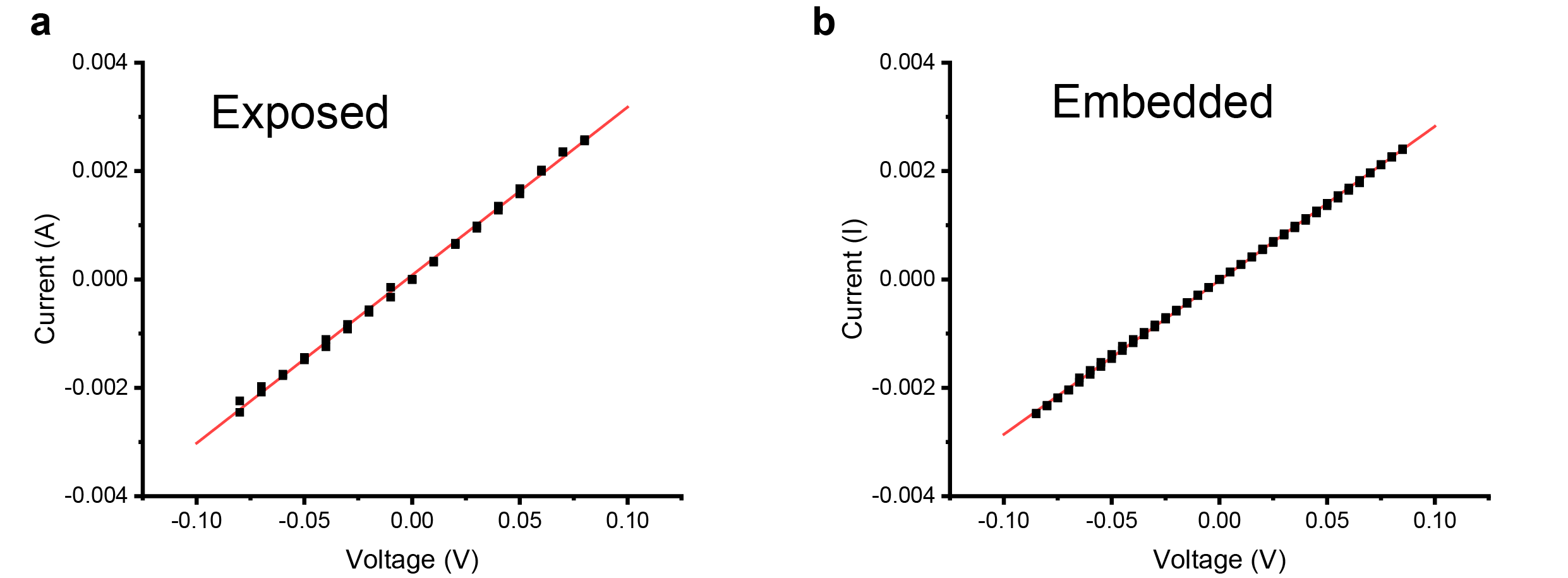


**Supplementary Figure S22. Electrical characterization of exposed and embedded tracks.** **a)** Linear fit of current-voltage (I-V) measurement on exposed track. Fit parameters: (slope = 0.031, R^2^ = 0.99806). **b)** Linear fit of current-voltage (I-V) measurement on embedded track. Fit parameters: (slope = 0.02842, R^2^ = 0.99966). Data points are reported as black squares and linear fit as continuous red line.


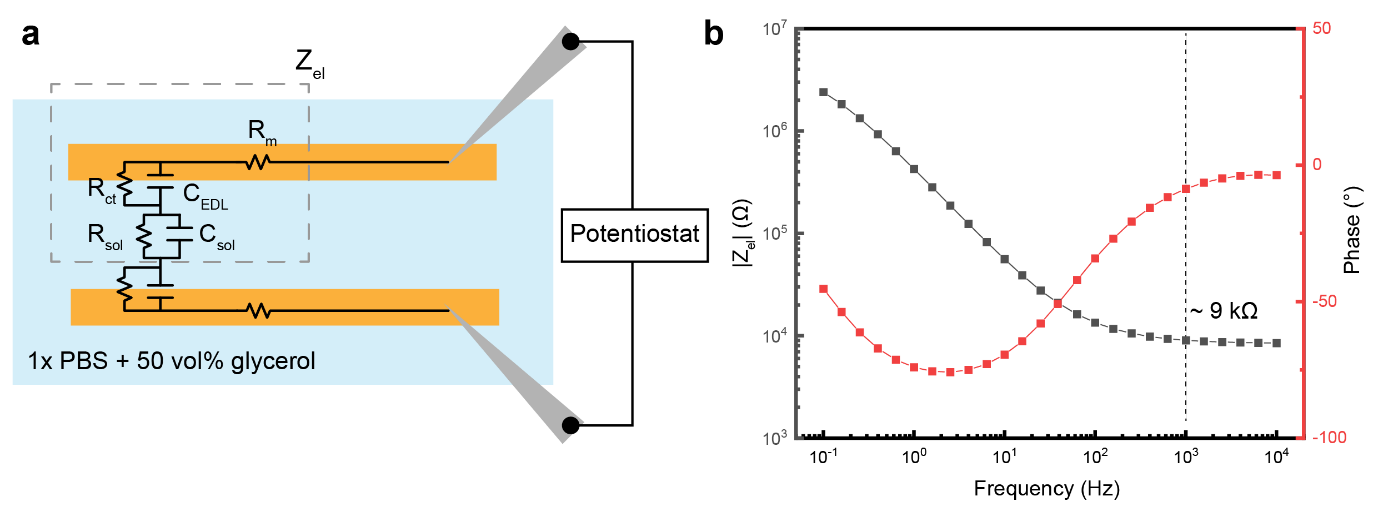


**Fig. S23. Estimation of thermal noise of patterned tracks. a)** Two-probe setup used for impedance spectroscopy and equivalent circuit. *R_m_*: resistance of the metal track; *R_ct_*: charge transfer resistance at the electrode-electrolyte interface; *C_EDL_*: capacitance of the double layer at the electrode-electrolyte interface; *R_sol_*: resistance of the solution contained within the hydrogel; *C_sol_*: geometrical capacitance of the hydrogel; *Z_el_*: total electrode impedance. **b)** Modulus and phase of *Z_el._* Dashed line indicates the impedance modulus at 1 kHz.

**Supplementary discussion of Fig. S23**

In bioelectronic recording, thermal noise is one of the major contributions to the total noise of the system. [4–7] In electrodes used, for example, as multi-electrode array, the equivalent thermal noise (*V_th,el_*) is introduced at the electrode-electrolyte interface. This noise can be computed as [4–6]

$$V_{th,el}=\sqrt{4k_{b}\cdot T\cdot Re(Z_{el})\cdot\Delta f}$$

Where *k_B_* is the Boltzmann constant, *T* is the absolute temperature, *Re(Z_el_)* is the real part of the electrode impedance (*Z_el_*), and *Δf* is the bandwidth.

To measure the electrode impedance, we conducted two-probe impedance spectroscopy using two parallel tracks patterned on the surface of a PDMAPS hydrogel. The hydrogel substrate was swollen in 1x PBS containing 50 vol% glycerol to reduce drying during the measurement. The system was modelled with an equivalent circuit shown in **Fig. S23**  where [8,9] *R_m,_* represents the resistance of the metal track; *R_ct_* the charge transfer resistance at the electrode-electrolyte interface; *C_EDL_*, the capacitance of the double layer at the electrode-electrolyte interface; *R_sol_*, the resistance of the solution contained within the hydrogel; and *C_sol_* the geometrical capacitance of the hydrogel. The equivalent circuit is considered symmetric on the lower track. The circuit schematic and measurement setup are shown in **Fig. S23a** and the impedance spectrum is shown in **Fig S23b**.

To estimate the contribution of the thermal noise in our system, we consider:

- *k_B_T* ~ 4.1∙10^-21^ J at 25°C
- *Re(Z_el_)* ~ 9 kΩ between 300 Hz and 5 kHz
- *Δf* : 300 Hz – 5 kHz, action potential region [5],

The equivalent thermal noise is *V_th,el_* ~ 0.8 µV. Considering *V_th,track_* the contribution to the thermal noise of the patterned metal track alone with *R_m_* ~ 30 Ω (as shown in **Fig. 4**), we obtain *V_th,track_* ~ 50 nV. Therefore, we expect the metal tracks to contribute minimally to the overall noise. However, due to the lack of a dielectric encapsulation layer between the tracks and the surrounding ionically-conductive hydrogel, we expect the inter-track impedance to be the major contribution to the thermal noise of the system.


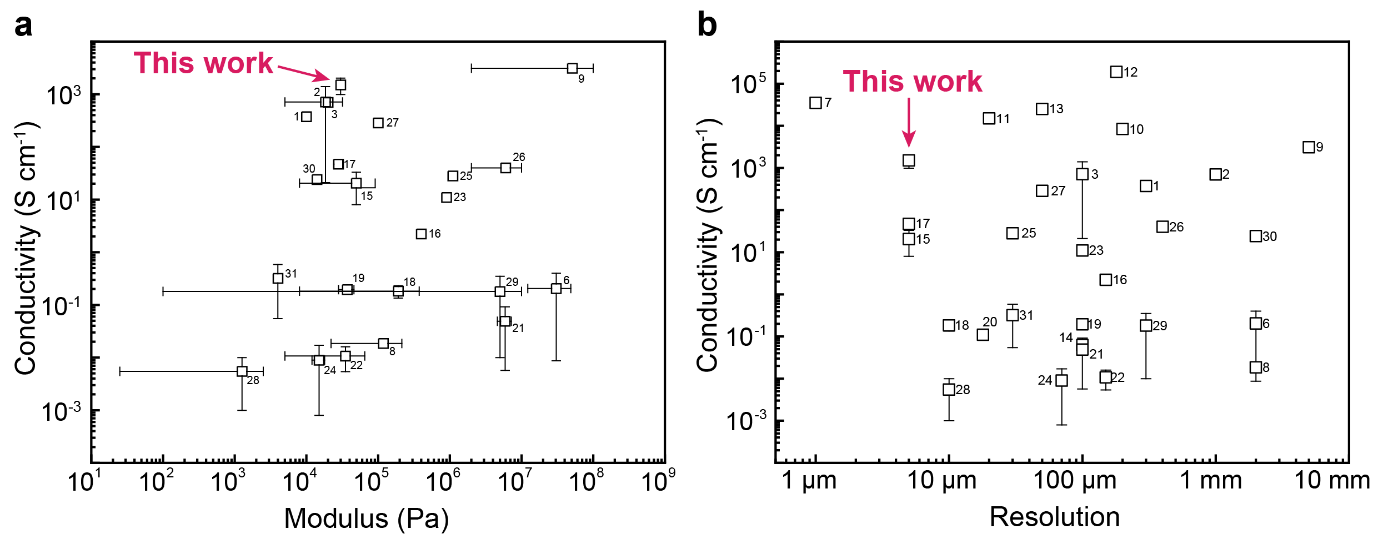
 **Supplementary Figure S24. Patternable and electronically conductive hydrogels**. References used for Ashby plots shown in **Fig. 6** in the main paper. Ashby plot of conductivity vs. **a)** modulus and **b)** resolution of conductive hydrogels. Data points are presented as mean ± standard deviation, whereas the cited work reported value ranges. Conductivity values are either directly taken from the referenced study or calculated from resistance measurements when the sample's geometrical parameters are available.

**Supplementary Table S1. Patternable and electronically conductive hydrogels.** References used for Ashby plots shown in **Fig. 6** in the main paper and in **Fig. S24**.

| Ref. number on plot | Reference |
| --- | --- |
| 1 | Yunsik Ohm et al., “An Electrically Conductive Silver–Polyacrylamide–Alginate Hydrogel Composite for Soft Electronics,” *Nature Electronics* 4, no. 3 (March 2021): 185–92, <https://doi.org/10.1038/s41928-021-00545-5> |
| 2 | Yongyi Zhao et al., “A Self-Healing Electrically Conductive Organogel Composite,” *Nature Electronics* 6, no. 3 (March 2023): 206–15, <https://doi.org/10.1038/s41928-023-00932-0>. |
| 3 | Yue Hui et al., “Three-Dimensional Printing of Soft Hydrogel Electronics,” *Nature Electronics* 5, no. 12 (December 2022): 893–903, <https://doi.org/10.1038/s41928-022-00887-8>. |
| 4 | Chanhyuk Lim et al., “Stretchable Conductive Nanocomposite Based on Alginate Hydrogel and Silver Nanowires for Wearable Electronics,” *APL Materials* 7, no. 3 (December 20, 2018): 031502, <https://doi.org/10.1063/1.5063657>. |
| 5 | Saurabh Awasthi and SeungYeon Kang, “Revolutionizing 3D Electronics: Single-Step Femtosecond Laser Fabrication of Conductive Embedded Structures and Circuitry,” *Materials Today Advances* 25 (March 1, 2025): 100544, <https://doi.org/10.1016/j.mtadv.2024.100544>. |
| 6 | Xiaowei Wang et al., “Stretch-Induced Conductivity Enhancement in Highly Conductive and Tough Hydrogels,” *Advanced Materials* 36, no. 25 (2024): 2313845, <https://doi.org/10.1002/adma.202313845>. |
| 7 | Pingping Zhao et al., “Formation of Silver Wires Embedded in Hydrogels Using Femtosecond Laser Ablation and Electroplating for Strain Sensing,” *Journal of Micromechanics and Microengineering* 32, no. 12 (November 2022): 125005, <https://doi.org/10.1088/1361-6439/ac9e63>. |
| 8 | Shohreh Azadi et al., “Biocompatible and Highly Stretchable PVA/AgNWs Hydrogel Strain Sensors for Human Motion Detection,” *Advanced Materials Technologies* 5, no. 11 (2020): 2000426, <https://doi.org/10.1002/admt.202000426>. |
| 9 | Kiyn Chin et al., “Accessible Soft Electronics with Silver-Gelatin Conductive Hydrogel Composite,” *Advanced Materials Technologies* n/a, no. n/a (2024): 2401193, <https://doi.org/10.1002/admt.202401193>. |
| 10 | Youngsang Ko et al., “A Simple Silver Nanowire Patterning Method Based on Poly(Ethylene Glycol) Photolithography and Its Application for Soft Electronics,” *Scientific Reports* 7, no. 1 (May 23, 2017): 2282, <https://doi.org/10.1038/s41598-017-02511-8>. |
| 11 | Yumi Ahn et al., “Highly Conductive and Flexible Silver Nanowire-Based Microelectrodes on Biocompatible Hydrogel,” *ACS Applied Materials & Interfaces* 6, no. 21 (November 12, 2014): 18401–7, <https://doi.org/10.1021/am504462f>. |
| 12 | Fengbo Zhu et al., “Integrated Multifunctional Flexible Electronics Based on Tough Supramolecular Hydrogels with Patterned Silver Nanowires,” *Journal of Materials Chemistry C* 8, no. 23 (June 18, 2020): 7688–97, <https://doi.org/10.1039/D0TC01011A>. |
| 13 | Tao Chen et al., “Fabrication of Three-Dimensional Metal Structures Embedded in Hydrogel by Using Femtosecond Laser Ablation and Electroplating,” *Optics Letters* 45, no. 22 (November 15, 2020): 6286–89, <https://doi.org/10.1364/OL.405854>. |
| 14 | Rikuto Miyakoshi, Shuichiro Hayashi, and Mitsuhiro Terakawa, “Direct Patterning of Conductive Structures on Hydrogels by Laser-Based Graphitization for Supercapacitor Fabrication,” *Advanced Electronic Materials* 9, no. 5 (2023): 2201277, <https://doi.org/10.1002/aelm.202201277>. |
| 15 | Wen Wang et al., “Photopatternable PEDOT:PSS Hydrogels for High-Resolution Photolithography,” *Advanced Science* n/a, no. n/a (2025): 2414834, <https://doi.org/10.1002/advs.202414834>. |
| 16 | Yuxin Liu et al., “Morphing Electronics Enable Neuromodulation in Growing Tissue,” *Nature Biotechnology* 38, no. 9 (September 2020): 1031–36, <https://doi.org/10.1038/s41587-020-0495-2>. |
| 17 | Yuxin Liu et al., “Soft and Elastic Hydrogel-Based Microelectronics for Localized Low-Voltage Neuromodulation,” *Nature Biomedical Engineering* 3, no. 1 (January 2019): 58–68, <https://doi.org/10.1038/s41551-018-0335-6>. |
| 18 | Vivian R. Feig et al., “Mechanically Tunable Conductive Interpenetrating Network Hydrogels That Mimic the Elastic Moduli of Biological Tissue,” *Nature Communications* 9, no. 1 (December 2018): 2740, <https://doi.org/10.1038/s41467-018-05222-4>. |
| 19 | Jaehyun Hur et al., “Polypyrrole/Agarose-Based Electronically Conductive and Reversibly Restorable Hydrogel,” *ACS Nano* 8, no. 10 (October 28, 2014): 10066–76, <https://doi.org/10.1021/nn502704g>. |
| 20 | Lijia Pan et al., “Hierarchical Nanostructured Conducting Polymer Hydrogel with High Electrochemical Activity,” *Proceedings of the National Academy of Sciences* 109, no. 24 (June 12, 2012): 9287–92, <https://doi.org/10.1073/pnas.1202636109>. |
| 21 | Dan My Nguyen et al., “One Pot Photomediated Formation of Electrically Conductive Hydrogels,” *ACS Polymers Au* 4, no. 1 (February 14, 2024): 34–44, <https://doi.org/10.1021/acspolymersau.3c00031>. |
| 22 | Jiawen Yu et al., “3D Printing of Robust High-Performance Conducting Polymer Hydrogel-Based Electrical Bioadhesive Interface for Soft Bioelectronics,” *Small* 20, no. 19 (2024): 2308778, <https://doi.org/10.1002/smll.202308778>. |
| 23 | Tao Zhou et al., “3D Printable High-Performance Conducting Polymer Hydrogel for All-Hydrogel Bioelectronic Interfaces,” *Nature Materials*, June 15, 2023, 1–8, <https://doi.org/10.1038/s41563-023-01569-2>. |
| 24 | Heng Zhu et al., “3D Printing of Conductive Hydrogel–Elastomer Hybrids for Stretchable Electronics,” *ACS Applied Materials & Interfaces* 13, no. 49 (December 15, 2021): 59243–51, <https://doi.org/10.1021/acsami.1c17526>. |
| 25 | Hyunwoo Yuk et al., “3D Printing of Conducting Polymers,” *Nature Communications* 11, no. 1 (March 30, 2020): 1604, <https://doi.org/10.1038/s41467-020-15316-7>. |
| 26 | Baoyang Lu et al., “Pure PEDOT:PSS Hydrogels,” *Nature Communications* 10, no. 1 (March 2019): 1043, <https://doi.org/10.1038/s41467-019-09003-5>. |
| 27 | Byungkook Oh et al., “3D Printable and Biocompatible PEDOT:PSS-Ionic Liquid Colloids with High Conductivity for Rapid on-Demand Fabrication of 3D Bioelectronics,” *Nature Communications* 15, no. 1 (July 11, 2024): 5839, <https://doi.org/10.1038/s41467-024-50264-6>. |
| 28 | Léo Sifringer et al., “Photopatterning of Conductive Hydrogels Which Exhibit Tissue-like Properties,” *Journal of Materials Chemistry B* 12, no. 40 (October 17, 2024): 10272–84, <https://doi.org/10.1039/D4TB00807C>. |
| 29 | Christina M. Tringides et al., “Viscoelastic Surface Electrode Arrays to Interface with Viscoelastic Tissues,” *Nature Nanotechnology* 16, no. 9 (September 2021): 1019–29, <https://doi.org/10.1038/s41565-021-00926-z>. |
| 30 | Yoonsoo Shin et al., “Low-Impedance Tissue-Device Interface Using Homogeneously Conductive Hydrogels Chemically Bonded to Stretchable Bioelectronics,” *Science Advances* 10, no. 12 (March 20, 2024): eadi7724, <https://doi.org/10.1126/sciadv.adi7724>. |
| 31 | Wan-Lou Lei et al., “All Biodisintegratable Hydrogel Biohybrid Neural Interfaces with Synergistic Performances of Microelectrode Array Technologies, Tissue Scaffolding, and Cell Therapy,” *Advanced Functional Materials* 34, no. 3 (2024): 2307365, <https://doi.org/10.1002/adfm.202307365>. |
| 32 | This work |


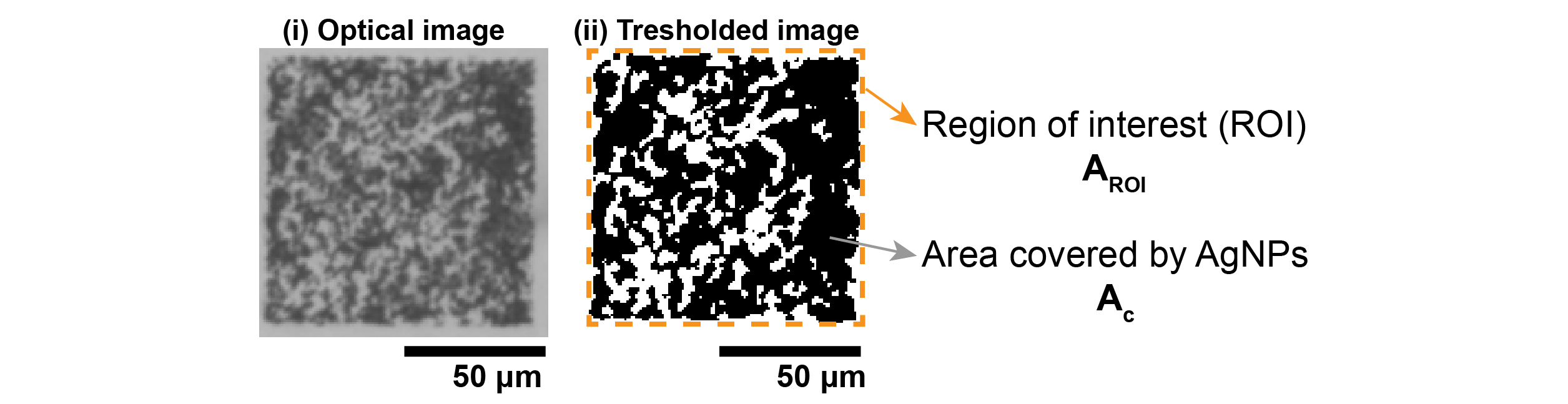


**Supplementary Figure S25. Surface coverage.** Example of optical micrograph of a square pattern (i) and corresponding thresholded image for surface coverage quantification (ii).

**References**

[1] H. Mohajer, M.R. Toosi, M.R. Zardoost, Appl. Surf. Sci. 615 (2023) 156391.

[2] S. Gürdap, N.N. Bayram, İ.A. İşoğlu, S. Dinçer İşoğlu, ACS Appl. Polym. Mater. 4 (2022) 6303–6311.

[3] M. Yao, Z. Wei, J. Li, Z. Guo, Z. Yan, X. Sun, Q. Yu, X. Wu, C. Yu, F. Yao, S. Feng, H. Zhang, J. Li, Nat. Commun. 13 (2022) 5339.

[4] M.D. Ferro, N.A. Melosh, Adv. Funct. Mater. 28 (2018) 1704335.

[5] V. Viswam, M.E.J. Obien, F. Franke, U. Frey, A. Hierlemann, Front. Neurosci. 13 (2019).

[6] D.A. Robinson, Proc. IEEE 56 (1968) 1065–1071.

[7] H. Yuk, B. Lu, X. Zhao, Chem. Soc. Rev. 48 (2019) 1642–1667.

[8] V.R. Feig, H. Tran, M. Lee, Z. Bao, Nat. Commun. 9 (2018) 2740.

[9] H. Yuk, J. Wu, X. Zhao, Nat. Rev. Mater. (2022) 1–18.
